# Response of bacterioplankton to iron fertilization of the Southern Ocean, Antarctica

**DOI:** 10.3389/fmicb.2015.00863

**Published:** 2015-08-26

**Authors:** Sanjay K. Singh, Arunasri Kotakonda, Raj K. Kapardar, Hara Kishore Kankipati, Pasupuleti Sreenivasa Rao, Pratibha Mambatta Sankaranarayanan, Sundareswaran R. Vetaikorumagan, Sathyanarayana Reddy Gundlapally, Ramaiah Nagappa, Sisinthy Shivaji

**Affiliations:** ^1^Council of Scientific and Industrial Research-Centre for Cellular and Molecular BiologyHyderabad, India; ^2^Council of Scientific and Industrial Research-National Institute of OceanographyGoa, India

**Keywords:** Southern Ocean, iron fertilization, metagenomic DNA, Major phylogenetic groups, unique bacterial clusters

## Abstract

Ocean iron fertilization is an approach to increase CO_2_ sequestration. The Indo-German iron fertilization experiment “LOHAFEX” was carried out in the Southern Ocean surrounding Antarctica in 2009 to monitor changes in bacterial community structure following iron fertilization-induced phytoplankton bloom of the seawater from different depths. 16S rRNA gene libraries were constructed using metagenomic DNA from seawater prior to and after iron fertilization and the clones were sequenced for identification of the major bacterial groups present and for phylogenetic analyses. A total of 4439 clones of 16S rRNA genes from ten 16S rRNA gene libraries were sequenced. More than 97.35% of the sequences represented four bacterial lineages i.e. *Alphaproteobacteria, Gammaproteobacteria, Bacteroidetes*, and *Firmicutes* and confirmed their role in scavenging of phytoplankton blooms induced following iron fertilization. The present study demonstrates the response of *Firmicutes* due to Iron fertilization which was not observed in previous southern ocean Iron fertilization studies. In addition, this study identifies three unique phylogenetic clusters LOHAFEX Cluster 1 (affiliated to *Bacteroidetes*), 2, and 3 (affiliated to *Firmicutes*) which were not detected in any of the earlier studies on iron fertilization. The relative abundance of these clusters in response to iron fertilization was different. The increase in abundance of LOHAFEX Cluster 2 and *Papillibacte*r sp. another dominant *Firmicutes* may imply a role in phytoplankton degradation. Disappearance of LOHAFEX Cluster 3 and other bacterial genera after iron fertilization may imply conditions not conducive for their survival. It is hypothesized that heterotrophic bacterial abundance in the Southern Ocean would depend on their ability to utilize algal exudates, decaying algal biomass and other nutrients thus resulting in a dynamic bacterial succession of distinct genera.

## Introduction

Oceans are a major source and sink for carbon (Coale et al., 1996) with the marine phytoplankton fixing up to 40% of the carbon dioxide (CO_2_) (Hassler et al., 2011). Thus factors that hinder CO_2_ fixation by marine phytoplankton would impact global climate due to increase in the levels of CO_2_ in the atmosphere. Both biotic (grazing of phytoplankton by microzooplankton) and abiotic factors (deficiency in the micronutrient iron) could decrease the levels of CO_2_ sequestered. Therefore the assumption is that if iron deficiency is overcome by exogenous addition of iron it would facilitate a phytoplankton bloom and thus lead to CO_2_ sequestration. The bloom could also be grazed by microzooplankton and excreted as fecal pellets. These two processes would bring about CO_2_ fixation (Smetacek and Naqvi, 2008).

Southern ocean, subarctic pacific, and eastern equatorial pacific waters are three major oceanic ecosystems known to be dominated by autotrophic pico- and nanoplanktons (Bradford-Grieve et al., 1999). Despite the dominance of these primary producers primary productivity is limited due to low iron concentration in spite of high nutrient concentration (Martin et al., 1994). Iron fertilization experiments of the surface seawater unequivocally demonstrated that iron is the limiting micronutrient for phytoplankton growth in these regions (Boyd et al., 2000, 2007; Gervais et al., 2002; Coale et al., 2004). The occurrence and persistence of the bloom following iron fertilization would depend on the micrzooplankton that graze on the phytoplankton and also on the bacterioplankton. Bacterioplankton intern depends on the phytoplankton for organic carbon produced. In pelagic ecosystems, bacterioplankton consume approximately 50% of primary productivity (Ducklow, 2000; Azam and Worden, 2004; Church, 2008) thus also implying that factors like temperature (Pomeroy and Deibel, 1986; Kirchman and Rich, 1997), dissolved organic matter (Kirchman, 1990; Kirchman and Rich, 1997), inorganic nutrients (Cotner et al., 1997) and micronutrients like iron (Pakulski et al., 1996; Church et al., 2000) which positively influence the growth of bacteria would have a negative impact on phytoplankton. In fact bacteria compete with phytoplankton for iron (Maldonado and Price, 1999) and the ability to synthesize siderophores, an iron scavenging system, gives bacteria an extra advantage over phytoplankton (Hutchins, 1995; Butler, 1998). In this cycle of CO_2_ fixation following iron fertilization of sea water a close association probably exists between iron availability, phytoplankton bloom and the abundance of microzooplankton and bacterioplankton i.e., the heterotrophic bacteria. In some of the earlier experiments, bacterial abundance, and heterotrophic production under conditions of iron fertilization was relatively low (Cochlan, 2001; Hall and Safi, 2001; Oliver et al., 2004; Suzuki et al., 2005). In such experiments the bacterial communities did not exhibit significant differences prior to and after iron fertilization (Hutchins et al., 2001a,b; Arrieta et al., 2004). In contrast, bacterial abundance, production, respiration (Blain et al., 2007; Obernosterer et al., 2007) and microbial succession had been observed during a phytoplankton bloom induced by natural iron fertilization on the Kerguelen Plateau (West et al., 2008).

To get a better insight into the bacteria-phytoplankton relationship in the context of iron fertilization, the aim of the present work was to analyse the effect of iron fertilization on bacterial community structure as part of the Indo-German iron fertilization experiment “LOHAFEX” (“Loha” means iron in Hindi and “Fex” is an acronym for fertilization) which was carried out in the Southern Ocean, Antarctica. This responsibility was divided between the German (Thiele et al., 2012) and the Indian teams (the present group). Thiele et al. (2012) as part of this work analyzed the bacterial community of the LOHAFEX samples by 454 tag pyrosequencing of 16S rRNA gene (422 ± 52 bp) and reported diversity analysis of clones affiliated to *Alphaproteobacteria, Gammaproteobacteria, and Bacteroidetes*. The majority of the clones could be identified up to the class, clade (*Roseobacter* clade, SAR 116 clade, SAR86, SAR92 clade etc.), family (*Rhodobacteraceae*) or group (marine) level and a few genera (*Balneatrix, Pseudospillum, Pelucidibaca, Pseudomonas, Formosa*, and *Ulvibacter*) were also identified. None of the clones could be identified up to the species level. Thiele et al. (2012) also studied changes in the bacterial community by CARD FISH using 11 oligonucleotide probes. Many of the probes were not specific like EUB 338 which detects most bacteria, ALP968 which detects *Alphaproteobacteria* except *Ricketsiales*, CF319a which detects most *Flavobacteria*, some *Bacteroidetes* and some *Sphingobacteria* and CF6-1267 which detect marine *Flavobacteria* including NS5 (See Table 1 of Thiele et al., 2012). Though this approach helped in quantifying the number of cells with respect to time and depth, it lacked specificity in relation to the identity of the bacteria. The results of this study demonstrated that small- celled bacterioplankton survived grazing pressure and were seen in response to Iron fertilization.

The present study was undertaken to identify bacterial community changes in the Southern Ocean waters, prior to and after iron fertilization, by sequencing the almost complete 16S rRNA gene (~1500 bp) sequence from clone libraries constructed using metagenomic DNA from water samples. This method is still a “gold standard” choice in microbial ecology, since the method uses suitable 16S rRNA gene size (~1500 bp), the available huge database for 16S rRNA gene make comparisons easy and identifies the sequences to species level. Thus this approach would help to identify the relative abundance and population dynamics of bacteria at the species level in response to iron fertilization. Alignment of the clones to the 454 pyrotag sequence region would also facilitate a comparison with the study of Thiele et al. (2012). In fact, this study identified in addition to classes *Alphaproteobacteria, Gammaproteobacteria*, and *Bacteroidetes* (Thiele et al., 2012) also clones affiliated to *Firmicutes, Nitrospinae, and Deinococcus-Thermus*. This study also identified three unique phylogenetic clusters LOHAFEX Cluster 1 (affiliated to *Bacteroidetes*), 2 and 3 (affiliated to *Firmicutes*) which were not detected in any of the earlier studies on iron fertilization. The increase in abundance of LOHAFEX Cluster 2 and *Papillibacter* sp. another dominant *Firmicutes* after iron fertilization implied a role for these taxa in phytoplankton degradation. The phylogenetic analyses provided valuable information on the identity of specific bacterial species in degradation of the phytoplankton bloom which was not possible in the earlier study.

## Materials and methods

### Description of sampling location and sampling

**Figure 1:**
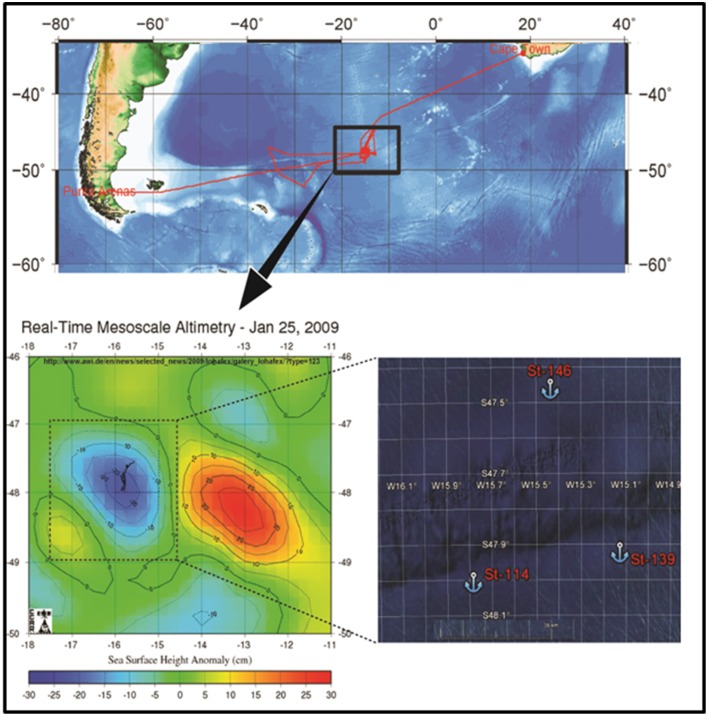
**Map showing the location of LOHAFEX experiment in the Southern Ocean waters (St-114, 48° 1.9758′ S and 15° 47.1336′ W and St-139 47° 57.1968′ S and 15)**.

The stable eddy which was fertilized was identified between 47–48° South and 15–17° West (Figure 1) in the Antarctic Circumpolar current (ACC). A total of 10 tons of dissolved ferrous sulfate was discharged over 150 km^2^ area of the sea. Water samples were collected from two locations during the experiment. The first sample was collected on day 1 just prior to fertilization from the in-patch (designated St-114, 48° 1.9758′ S and 15° 47.1336′ W) and the second sample was collected on day 14 after fertilization from the in-patch (designated St-139 47° 57.1968′ S and 15 8.622′ W). Seawater was collected using a Niskin sampler (General Oceanic, USA) from surface (~0–3 m), 40 m (~chlorophyll maxima), 100, 300, and 500 m depth. Water samples (~2L) were filtered through 0.22 μm polycarbonate filters (45 mm, Millipore, U.S.A) and the filters were stored at −80°C for the extraction of DNA. Here after samples from St-114 and St-139 would be referred to as unfertilized water mass and fertilized water mass respectively.

### Ethics statement

The scientific experiment is in accordance with the resolution of the London Convention on the regulation of ocean fertilization from October 2008 and the Decision of the Convention on Biological Diversity on ocean fertilization from May 2008 that call for further research to enhance understanding of ocean iron fertilization. For LOHAFEX, the National Institute of Oceanography (NIO), India, and the Alfred Wegener Institute for Polar and Marine Research in the Helmholtz Association, Germany, have evaluated possible environmental impacts showing that this experiment will not cause damage to the environment. The level to which the surface-water iron concentrations will be enhanced during this experiment is an order of magnitude lower than natural iron levels in coastal marine waters. This concentration is so low that most analytical laboratories in the world cannot measure it. The fertilized waters, although located offshore, have been previously in contact with the coast of South America and South Georgia and contain coastal plankton species that are adapted to high iron concentrations. The size of the fertilized patch is considerably smaller than the impact of melting icebergs that may leave a swath of several hundred kilometers breadth of enhanced iron concentrations.

LOHAFEX is a contribution to POGO, the Partnership for Observation of the Global Oceans (http://www.ocean-partners.org).

### Total prokaryotic cell count

To ascertain the total prokaryotic cell count (TPC) 25 ml of seawater from unfertilized and fertilized water masses collected from surface (0–3 m), 40, 100, 300 and 500 m depths were preserved in 2% (v/v) buffered formaldehyde. Subsequently, the formaldehyde preserved seawater samples were stained with 4′6′-diamidino-2-phenylindole (DAPI; 1 μgml^−1^, final concentration), filtered through 0.22 μm black nuclepore membrane and counted under a fluorescent microscope as described by Hobbie et al. (1977).

### Chlorophyll *a* analysis

The estimation of chlorophyll *a* was done by fluorometer (Turners Design 10AU, USA) method (Banse, 1988). Chlorophyll *a* was estimated up to 100 m depth only.

### Estimation of bacterial production

Bacterial production was estimated based on the incorporation rates of [methyl-^3^H] thymidine (^3^H-TdR) and Leucine (^3^H-Leu). For ^3^H-TdR three replicates of 20 ml water samples were incubated at 5°C for 3 h with 100 μl of 60 nM working solution of ^3^H-TdR (specific activity 18,000 mCi mM^−1^; Amersham Corp., Amersham, England). The thymidine uptake reaction was stopped with 300 μl formaldehyde, bacterial cells collected on 0.22 μm cellulose acetate filters (Millipore India Ltd, Bangalore, India) and the filter papers were rinsed in cold trichloroacetic acid (TCA; 10% w/v) and ethanol (96% v/v; final rinse). The filters were transferred to 8 ml scintillation vials containing 5 ml scintillation cocktail Ultima Golds RX (PerkinElmer, Waltham, USA) and radioactivity counts were determined in a liquid Scintillation Counter (Perkin Elmer, Waltham, USA). The amount of incorporated thymidine (^3^H-TdR, pM l-1 h-1) was calculated using the formula given by Fuhrman and Azam (1980).

^3^H-Leu incorporation assay was done in 2 ml centrifuge tubes. To 2 ml of water sample 0.05 nM ^3^H-Leu (specific activity 50,000 mCi mM-1; Amersham Corp.,7Amersham, England) was added and incubated at 5°C for 1.5 h. All the incubations were stopped with 5% TCA. Cells were pelleted by centrifugation (16,000 rpm for 10 min). Ultima Golds RX scintillation cocktail was added to the microcentrifuge tubes and the samples counted in a scintillation counter. The details of the method have been described by Smith and Azam (1992). Formaldehyde fixed samples prior to the addition of ^3^H-TdR or ^3^H-Leu served as a control.

### Extraction of DNA from water samples, amplification of 16S rRNA gene, construction of 16S rRNA gene libraries and sequencing

Metagenomic DNA was extracted from the filters as described previously (Shivaji et al., 2004). In brief, filters were cut into small pieces, using a sterile blade and suspended in a sterile 15 ml centrifuge tube containing 500 μl of lysis buffer (0.15 M NaCl, 0.1 M EDTA, pH 8) with lysozyme (final concentration 30 mg ml^−1^). Tubes were incubated at 37°C for 2 h. After that 25 μl each of SDS (20% stock) and proteinase K (10 mg ml^−1^) were added to the tubes and incubated at 55°C for 1 h. The contents were extracted twice with phenol: chloroform: isoamyl alcohol (25:24:1) and chloroform and isoamyl alcohol (24:1). The aqueous phase was separated and DNA was precipitated with sodium acetate and isopropanol. The precipitated DNA was recovered by centrifugation (15000 g), washed twice with 70% ethanol and dissolved in 10 mM Tris-HCl (pH 8). The concentration and integrity of DNA was checked by Nanodrop (Nano Drop Technologies, USA) and agarose gel electrophoresis respectively.

Approximately, 10–100 ng of DNA was used for PCR amplification of 16S rRNA gene using bacteria-specific 16S rRNA gene universal primers PA (5′-AGAGTTTGATCCTGGCTCAG-3′) and PH (5′-AAG GAGGTGATCCAGCCGCA-3′) which amplify ~1500bp of bacterial 16S rRNA gene (Edwards et al., 1989). The temperature profiles for this amplification was as follows: initial denaturation at 95°C for 5 min followed by 25 cycles of 94°C for 1 min, 55°C for 1 min, 72°C for 1 min and a final elongation step at 72°C for 10 min. The tube without DNA was used as negative control. For each sample minimum of 4 replicates of amplification reactions were carried out. Replicate samples were pooled and purified using QIAquick PCR purification kit (Qiagen, USA). The PCR products were cloned in pGEM-T easy cloning vector (Promega, USA) for 14 h at 4°C. The ligation mixture was transformed in to electrocompetent *E. coli* DH5α cells. Transformants were selected based on blue white selection on LB agar plates containing ampicillin (100 μgml-1), Xgal (40 μgml-1) and IPTG (0.1 mM). In all, 10 libraries were constructed corresponding to the two stations (St-114 and St-139). The libraries were named as Station number-depth (for example St-114-sur for surface water sample at St-114).

White colonies were picked up randomly and the 16S rRNA gene was amplified from the transformants by colony PCR using the vector-targeted M13 forward (5′-GTA AAA CGA CGG CCA GT-3′) and M13 reverse (5′-GGA AAC AGC TAT GAC CAT G-3′) primers, respectively, and sequenced using the primers M13 forward, M13 reverse, pD (5′-CAG CAG CCG CGG TAA TAC-3′) and pF^*^ (5′-ACG AGC TGA CGA CAG CCA TG-3′) (Pradhan et al., 2010). Sequencing reaction was performed using Big dye terminator v3.1 kit (Applied Biosystems, U.S.A) as per manufacturer instruction and run on ABI 3130XL. The sequences were assembled using Auto Assembler sequence assembly software (Applied Biosystems, U.S.A) and vector sequences were removed manually. Approximately 500 positive clones from each library were sequenced for almost full length 16S rRNA gene sequence.

### Phylogenetic and statistical analysis of the libraries

Mallard program was used to check for chimera (Ashelford et al., 2005). All the bad quality sequences (*n* = 207), short sequences (*n* = 235) and chimeras (*n* = 119), were removed prior to analysis. Sequences were named using a 10 letter code where the first letter was C, the next three digits refer to the identity of the specific station (e.g., 114 and 139), next three digits for depth (e.g., 001 = surface/~0 m, chl = chlorophyll maxima/~40 m, 100 = 100 m, 300 = 300 m, and 500 = 500 m) and the last three digits were for the clone number (e.g., 001–499). All non-chimeric sequences obtained from the 10 libraries were aligned together using MOTHUR program (Schloss et al., 2009). Sequences having similarity = 97% similarity were considered as belonging to the same operational taxonomic unit (OTUs). Only one representative sequence from each OTU was used to construct phylogenetic trees. Representative OTUs were checked for similar sequences present in Silva data base (http://www.mothur.org/wiki/Silva_reference_files) and downloaded. All representative OTUs and their similar sequences were aligned with MOTHUR and the alignment was edited manually to check for ambiguous alignment. The phylogenetic trees were constructed using neighborhood joining method available in MEGA5 (Tamura et al., 2011). The evolutionary distances among the sequences were calculated using maximum likelihood method (Tamura and Nei model) available in MEGA5 (Tamura et al., 2011). The reliability of the phylogenetic tree was checked by bootstrap analysis (1000 times).

All the statistical analyses were carried out at 97% sequence similarity level (0.03 distances). The Shannon diversity index (H'), rarefaction analysis, non-parametric estimation of diversity (Chao I), Evenness and Good's coverage were calculated for all the libraries using MOTHUR. Statistical comparisons of the libraries were carried out using Unifrac (Lozupone and Knight, 2005). In Unifrac analysis, different bacterial groups from all depths were compared separately for stations 114 and 139. The normalized Unifrac abundance test was used for bacterial group comparison. To compare the percentage relative abundance of bacterial groups between different stations, 95% upper and lower binomial central confidence were calculated from online tool available at http://www.causascientia.org/math_stat/ProportionCI.html. Two by two Fischer's test was used to compare the statistical difference between the abundance of the OTUs of each bacterial group from stations 114 and 139. (http://www.quantpsy.org/fisher/fisher.htm). Heat map was derived for the relative abundance data at various depths using “CIMminer” online software (http://discover.nci.nih.gov/cimminer/).

### Mapping of sanger sequences with the 454 reads

The 16S rDNA clone library Sanger sequences of the present study and the 454 reads of Thiele et al. (2012) were analyzed independently using “MOTHUR” to obtain the operational taxonomic units (OTUs) at 97% threshold limit (Schloss et al., 2009). One representative sequence from each OTU was used for mapping the Sanger data with that of 454 data in two different approaches viz., hierarchal taxonomic mapping and phylogenetic mapping. The taxonomic distinction between the two data sets was revealed by comparing and mapping of representative sequences using Ribosomal data base project (RDP) library compare web tool (https://rdp.cme.msu.edu/comparison/comp.jsp). This resulted in hierarchical mapping of number of representative sequences at various taxonomic levels. For phylogenetic mapping analysis 454 read representative sequences of 200–450 bp length were aligned with 1500 bp length Sanger sequences using ClustalX2 (Larkin et al., 2007) and only the aligned region was considered for bootstrap N-J tree in ClustalX2. Unaligned regions were manually deleted.

## Results

### Chlorophyll *a* concentration, total prokaryotic cell count, and bacterial production

**Figure 2:**
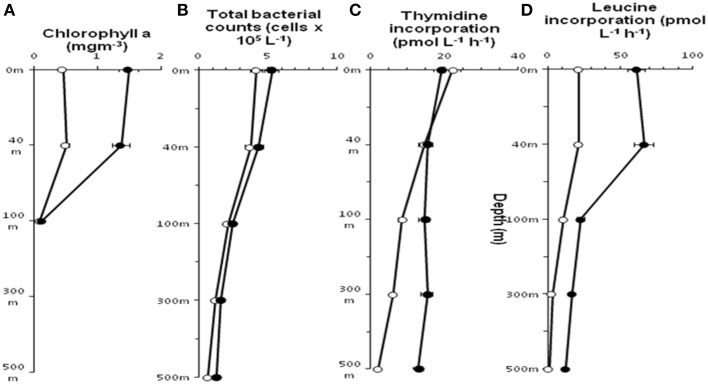
**Distribution of Chlorophyll ***a*** (A), Total bacterial count (B), incorporation of Thymidine (C) and Leucine (D) in stations St-114(O) and St-139 (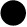) at different depths (0–3, 40, 100, 300, and 500 m)**.

Chlorophyll *a* concentration at St-139 up to 40 m depth was >3 times (*p* < 0.001) compared to the concentrations at St-114 indicating that iron fertilization has caused an increase in chlorophyll containing organism. At St-139, the chlorophyll *a* ranged between 0.13 and 1.58 mg m^−3^ while it was 0.10 and 0.75 mg m^−3^ in the non-fertilized water columns (Figure 2A).

In general, both stations showed a decrease in mean total prokaryotic cell count (TPC) with depth (Figure 2B) and TPC in the fertilized waters was slightly higher compared to non-fertilized station. At both the stations thymidine (^3^H-TdR) incorporation at the surface and up to 40 m depth was very similar (Figure 2C). But, subsequently distinct increase in ^3^H-TdR incorporation was observed up to 500 m in the fertilized water mass compared to the control station i.e., St-114. Leucine (^3^H-Leu) incorporation ranged between 1.1 and 66.6 pmol L^−1^ h^−1^ (Figure 2D) and the incorporation rate was 2–11.5 fold higher in iron fertilized water compared to the control station. A maximum of 11.5 fold change was observed at 500 m depth between St-139 and St-114.

### Bacterial community composition of sea water based on OTUs

The total bacterial community composition of the unfertilized and fertilized sea water mass was ascertained using a total of ten 16S rRNA gene libraries (five libraries from each of the two stations St-114 and St-139). Water samples were collected at each station from a particular depth i.e., surface (~0–3, 40, 100, 300, and 500 m respectively) for the construction of the 10 libraries. The 10 libraries yielded a total of 4439 sequences of 16S rRNA gene. All these sequences have been submitted to Genbank data base under accession numbers JX524894 to JX529332. Based on sequence similarity, the 4439 sequences could be categorized into 277 operational taxonomic units (OTUs) (Table S1) with members of an OTU exhibiting greater than 97% sequence similarity. The analyses also revealed that 137 out of the 277 OTUs were singletons (Table S1). The predominant OTUs (>100 sequences per OTU) include OTU50 (534), OTU97 (501), OTU112 (405) OTU169 (210), OTU191 (117), OTU208 (214), OTU218 (388), and OTU274 (526) (2895 sequences) (Table S1).

### Bacterial community composition of sea water based on different classes and genera of bacteria

**Figure 3:**
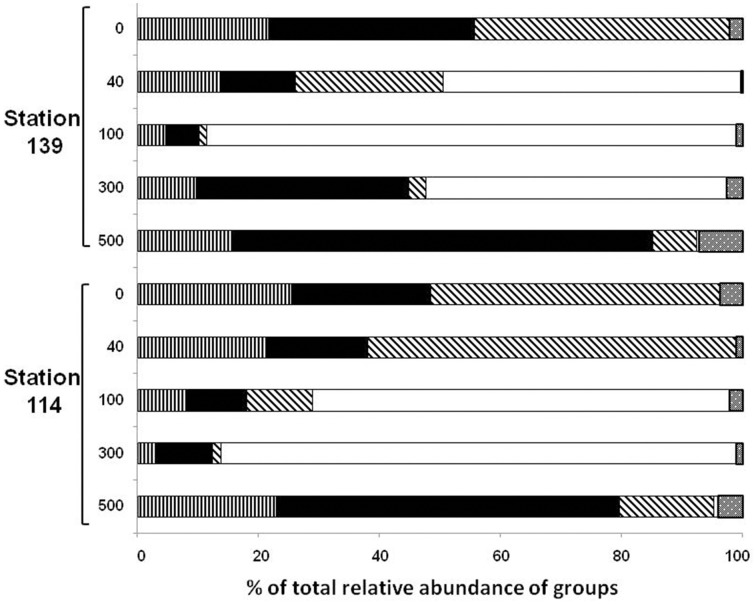
**Relative abundance (%) of clones affiliated to ***Alphaproteobacteria*** (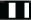), ***Gammaproteobacteria*** (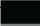), CFB (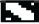), ***Firmicutes*** (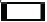), and others (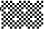) in the ten 16S rRNA gene libraries constructed using metagenomic DNA from seawater from five different depths (0–3, 40, 100, 300, and 500 m)**.

**Figure 4:**
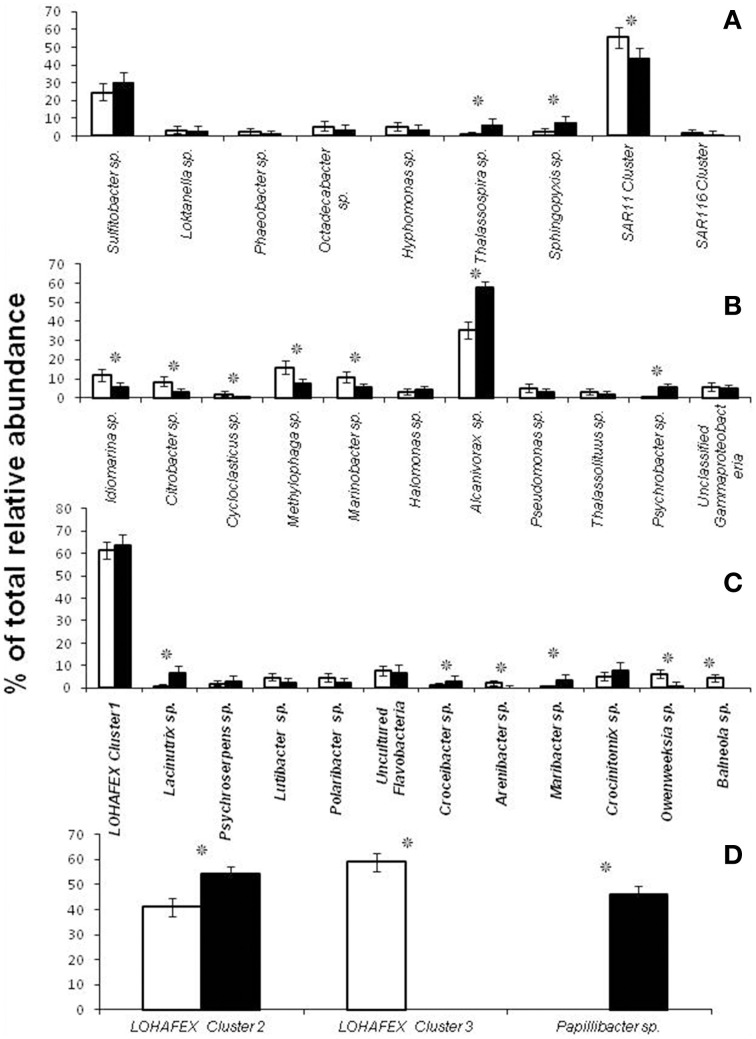
**Relative abundance (%) of clones affiliated to different genera of ***Alphaproteobacteria*** (A), ***Gammaproteobacteria*** (B), ***Bacteroidetes*** (C) and ***Firmicutes*** (D) in stations St-114 (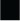) and St-139 (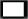)**. Asterisk indicates significant increase or decrease (***p*** < 0.05) prior to after fertilization.

The total diversity of the bacterial community in the sea water masses from five different depths, was ascertained by analysing the ten 16S rRNA gene libraries at the class and genera levels (Figures 3, 4). Taken together, 97.35% of the sequences in the 10 libraries were affiliated to three bacterial classes and one phylum i.e., *Alphaproteobacteria, Gammaproteobacteria, Bacteroidetes*, and *Firmicutes*. The remaining sequences (2.65%) were related to *Betaproteobacteria, Deltaproteobacteria*, cyanobacteria/plastid sequence, *Actinobacteria, Nitrospinae, Planctomycetes, Verrucomicrobia*, and *Deinococcus-Thermus* (Figures 3, 4 and Figures S1A–E).

### Phylogenetic analysis of the clones affiliated to different classes

Phylogenetic trees were constructed using sequences from all the 277 OTUs and with their related sequences which were downloaded from NCBI Nucleotide Data base (Figures S1A–E).

#### Alphaproteobacteria

Sequences related to *Alphaproteobacteria* constituted 14.69% of the total clones and it included SAR11 (20 OTUs with 281 clones), SAR116 (4 OTUs with 7 clones) and *Sulfitobacter sp*. (4 OTUs with 145 clones) (Figure S1A). OTU169 with 210 clones was the most abundant OTU in SAR11 cluster and the sequences exhibited 100% similarity with Candidatus *Pelagibacter ubique*. The *Sulfitobacter* cluster is the next most abundant genus in the *Alphaproteobacteria* (Figure S1A) with clones affiliated to *S. Mediterraneus* (52 clones) and *S. dubius* (93 clones) respectively (Figure S1A). The other clones of *Alphaproteobacteria* were affiliated to the genera *Loktanella* (16 clones), *Pelagicola* (2 clones), *Phaeobacter* (10 clones), *Octadecabacte*r (25 clones), *Litoreibacter* (38 clones), *Hyphomonas* (24 clones), *Thalassospira* (18 clones), and *Sphingopyxis* (26 clones). All the above clones were affiliated to a known cultivable species from the genus (Figure S1A).

#### Gammaproteobacteria

Sequences related to *Gammaproteobacteria* constituted 27.3% of the total clones. Five of the Gammaproteobacterial OTUs (OTU26, 143, 145, 256, and 274) representing 534 sequences showed affiliation with *Alcanivorax borkumensis* (528 sequences) and—*Alcanivorax dieselolei* (Figure S1B)*. Four* other OTUs (OTU002, 034, 078, and 191) were phylogenetically similar to *Methylophaga* sp. JAM1 (118 clones) (Figure S1B). One OTU (OTU033with 83 clones) was closely related to *Marinobacter algicola* (Figure S1B). Many more OTUs were related to known species such as *Citrobacter freundii* (OTU110), *Halomonas variabilis* (OTU174), and *Pseudomonas alcaligenes* (OTU10, 150, 185, and 245) (Figure S1B) and others identified with the genera *Idiomarina, Thalassolituus, Psychrobacter*, and unculturable bacterial clones (Figure S1B).

#### Bacteroidetes

**Figure 5:**
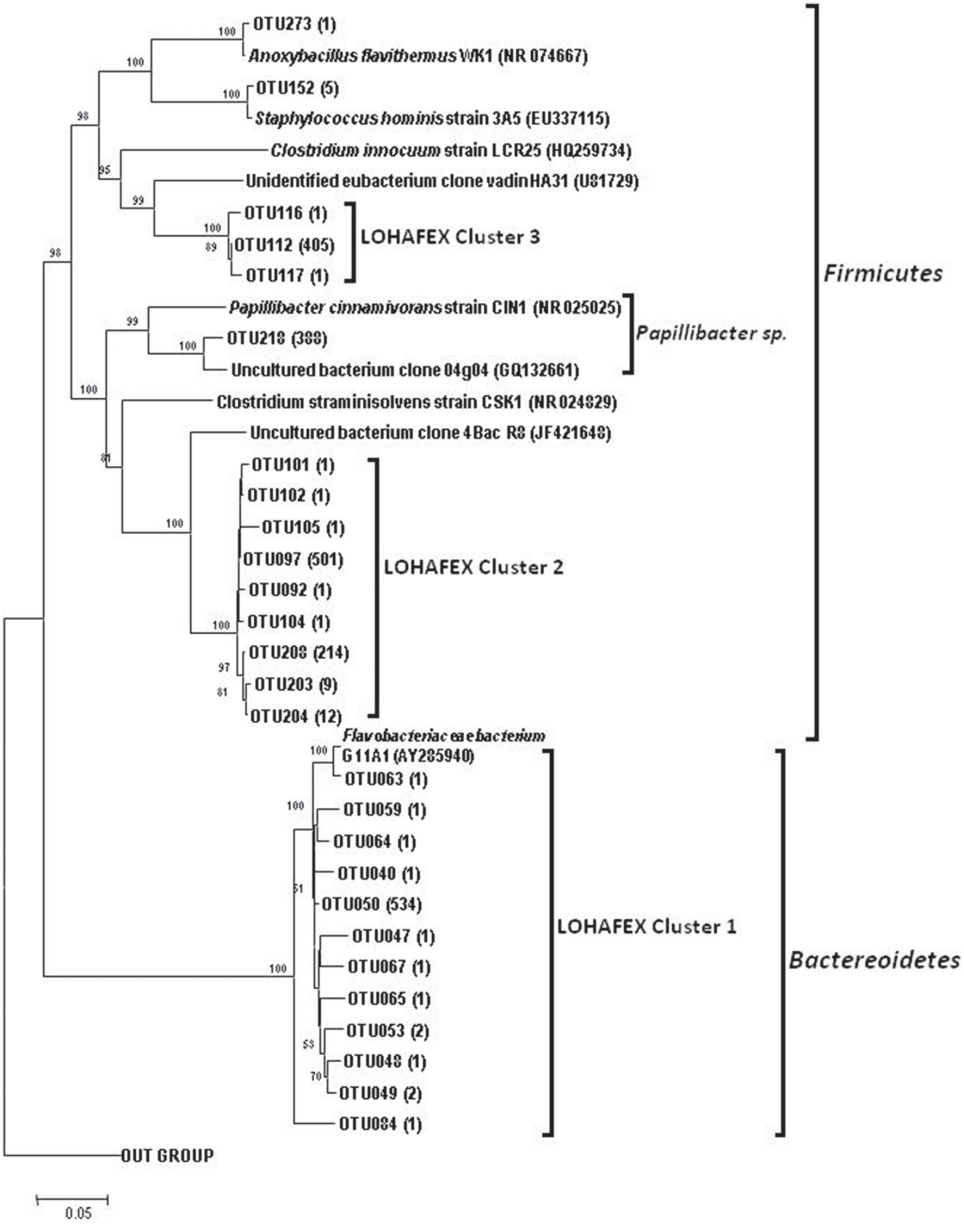
**Neighbor joining phylogenetic tree of 16S rRNA gene clones from 10 libraries of stations St-139 and St-114, showing the phylogenetic relationship of clones affiliated to ***Firmicutes*** and Lohafex cluster**. OTUs that showed more than 10 clones affiliated to a group of bacteria were considered for tree construction. *Agrobacterium larrymoorei* 3-10^T^ (Z30542) was taken as an out-group. Numbers at nodes are bootstrap values. The bar represents 0.05 substitutions per alignment position.

OTU050 (534 clones) was the most abundant OTU in *Bacteroidetes* and it formed a clade along with 10 other singleton OTUs (Figures S1C,D). This cluster with 547 clones was very different from the sequences in Silva DATA base or Gen Bank Data base and formed a unique clade affiliated to *Flavobacteriaceae* bacterium G11A1 and was also related to cultivable species such as *Lacinutrix algicola, Psychroserpens burtonensis*, and *Winogradskyella rapida* (Figure S1C). This unique cluster was designated as LOHAFEX Cluster 1 (Figures S1–D and Figure 5). The remaining OTUs in *Bacteroidetes* were affiliated to cultivable species of *Lutibacter, Polaribacter, Kordia, Croceibacter*, Arenibacter, *Maribacter, Crocinitomix, Owenweeksia, and Balneola* (Figure S1C). Seven OTUs were also closely related to uncultured *Flavobacteria* (Figure S1C).

#### Firmicutes

The *Firmicutes* represented 34.73% clones and belonged to 15 OTUs. Phylogenetic analysis of the sequences showed monophyletic origin of 9 OTUs which formed a clade with a distinct monophyletic origin. OTU097 (501 clones) and OTU208 (214 clones) were not affiliated to any known *Firmicutes* and were thus designated as LOHAFEX Cluster 2 (Figure 5). The closest phylogenetic neighbor of this clade was an uncultured bacterium clone 4Bac R8 which is distantly related to *Clostridium straminisolvens* (Figure 5). Three other OTUs 112 (405 clones), 116 (1 clone) and 117 (1 clone) were affiliated to *Firmicutes* but formed a separate clade distinct from LOHAFEX Cluster 2 and was designated as LOHAFEX Cluster 3 and was related to a bacterial clone vadin HA31. LOHAFEX Cluster 3 is distantly related to *Clostridium innoculum* (Figure 5). Clones affiliated to *Firmicutes* also included OTU218 (388 clones) which was closely related to uncultured bacterium clone 04g04 and distantly related to *Papillibacter cinnamivorans* (Figure 5). Two OTUs were related to *Staphylococcus hominis* and *Anoxybacillus flavithermus* respectively (Figure 5 and Figure S1D).

### Other bacterial groups

A total of 20 clones could be grouped into 10 representative OTUs related to *Actinobacteria*. In addition OTUs related to *Deltaproteobacteria, Betaproteobacteria, Cyanobacteria, Planctomycetes, Verrucomicrobia, Nitrospinae*, and *Deinococcus-Thermus* were also detected represented by 4, 5, 1, 4, 4, 3, and 1 OTUs respectively. The number of clones in each of these OTUs ranged from 1 to 19 (Figure S1E).

### Statistical analysis of the libraries

The number of OTUs varied among the libraries (Table 1). The Chao 1 values ranged between 39 and 180. The mean Good's coverage ranged from a minimum of 88.56% for St114-Chlmax and to a maximum of 96.95% for St-139-Chlmax, which indicated that all the libraries were sampled well in this study. Shannon diversity index values ranged from 0.86 to 3.34. In general, H' was higher in water column up to 40 m depth than 100 m and 300 m depths. Further, there is an increase in H' at 500 m depth. H' showed that diversity was higher before iron fertilization (St-114) compared to after iron fertilization (St-139).

**Table 1:** **Diversity indices for the ten 16S rRNA clone libraries constructed using metagenomic DNA from seawater from different depths (0–3, 40, 100, 300, and 500 m) of stations St-114 and St-139**.

**Stations**	**Depth**	**Similarity**	**Total number of clones**	**Observed OTUs**	**Shannon (H')**	**Evenness**	**Good's Coverage (%)**	**Chao1**
114	Surface (0 m)	0.97	487	83	3.34	0.71	93.02	111
	Chlmax (40 m)	0.97	402	76	3.18	0.67	88.56	180
	100 m	0.97	411	48	1.98	0.45	91.97	136
	300 m	0.97	478	32	1.04	0.25	96.65	44
	500 m	0.97	436	75	3.59	0.79	92.66	116
139	Surface (0 m)	0.97	411	65	3.27	0.75	93.67	98
	Chlmax (40 m)	0.97	426	38	2.24	0.58	96.95	46
	100 m	0.97	445	24	0.86	0.22	96.85	39
	300 m	0.97	499	51	2.28	0.54	95.99	68
	500 m	0.97	444	58	2.70	0.63	94.82	86

Results from rarefaction analysis (Figure S2) showed asymptotic plateau except at St114-40 m. The libraries from iron fertilized water columns reached to asymptotic plateau earlier than the non-fertilized water column. The Unifrac analysis of libraries indicated that the phylogenetic structure of the bacterial community composition (beta diversity of bacterial community) was stable following iron fertilization at all depths for different bacterial groups (*P* > 0.001 for all bacterial groups of station 114 and station 139 depths comparison).

### Effects of iron fertilization on bacterial community composition of sea water at different depths

#### Dynamics of *Alphaproteobacteria*

The relative abundance of *Alphaproteobacteria* was higher in the upper water column (up to 40 m) compared to 100–300 m depth in St-114 and St-139. In both the stations the abundance recovered at 500 m depth and was similar to levels observed at the surface. Maximum abundance of *Alphaproteobacteria* was observed to be similar in St-114 at the surface and at 40 m depth (Figure 3).

Depth profiling of the various genera of *Alphaproteobacteria* prior to and after fertilization with iron did reveal certain similarities and differences. For instance clones affiliated to SAR11 cluster were predominant at the surface and decreased with depth prior to and after iron fertilization, SAR116 clones were detected only up to a depth of 40 m and SAR406 clones appeared only at depths greater than 100 m and that to only in the presence of iron (Table 2). Clones affiliated to *Thalassospira sp*. and *Sphingopyxis sp*. increased in relative abundance after iron fertilization whereas clones affiliated to *SAR11 Cluster* decreased in relative abundance (Figure 4A and Table 2).

**Table 2:** **Relative abundance (%) of clones affiliated to different genera of ***Alphaproteobacteria, Gammaproteobacteria, Bacteroidetes*** and ***Firmicutes*** in stations St-114 and St-139 at different depths**.

**Taxa**	**114–5 m**	**114–40 m**	**114–100 m**	**114–300 m**	**114–500 m**	**139–5 m**	**139–40 m**	**139–100 m**	**139–300 m**	**139–500 m**
***ALPHAPROTEOBACTERIA***										
*Sulfitobacter* sp.	2.3	7.5	2.0	1.0	6.3	7.1	3.6	0	3.4	5.4
*Loktanella* sp.	0	1.4	0.5	0.2	0.3	0	0.2	0	0.4	1.0
*Phaeobacter* sp.	0.2	1.4	0.2	0	0	0	0	0	0.6	0
*Octadecabacter* sp.	0.9	1.9	0	0	1.4	1.0	1.2	0	0	0
*Hyphomonas* sp.	0.9	0	0.2	0.2	2.5	0	0	0	1.1	1.0
*Thalassospira* sp.	0	0.3	0.3	0	0	0.5	0	0.5	1.7	1.0
*Sphingopyxis* sp.	1.1	0	0	0	0.3	0.3	0	0	0	4.9
SAR11 Cluster	16.9	10.9	3.6	1.3	9.0	13.3	6.5	4.0	2.1	2.8
SAR116 Cluster	0.7	0.5	0	0	0	0.3	0.2	0	0	0
***GAMMAPROTEOBACTERIA***										
*Idiomarina* sp.	5.0	1.7	3.1	0.4	2.5	5.8	1.2	0.5	0.2	2.0
*Citrobacter* sp.	0.9	1.4	0.5	0	6.8	0.8	0.5	0.2	0.9	3.1
*Cycloclasticus* sp.	0.4	0	0	0	1.9	0.5	0	0	0.2	0
*Methylophaga* sp.	5.0	0.8	1.3	0.4	10.4	4.2	4.3	0.2	2.6	1.0
*Marinobacter* sp.	0.9	0	0.3	1.7	9.3	1.0	0	0	4.5	2.8
*Halomonas* sp.	0.2	0.3	0	0.2	2.5	1.0	0.2	0.7	2.1	2.3
*Alcanivorax* sp.	3.6	6.4	3.4	3.9	23.3	8.5	4.3	3.2	20.9	56.0
*Pseudomonas* sp.	0.7	0.8	0	0.4	3.8	0.8	0.5	0	1.3	2.3
*Thalassolituus* sp.	2.3	0.6	0	0	0	2.9	0	0	0	0.5
*Psychrobacter* sp.	0	0.3	0	0	0	5.3	0	0	0.9	3.3
*Unclassified Gammaproteobacteria*	1.6	2.2	0.3	0.8	1.4	1.3	0.7	0.2	0.6	2.8
***BACTEROIDETES***										
LOHAFEX Cluster1	39.5	41.0	7.6	0.4	1.1	30.2	21.2	0	0	0
*Lacinutrix* sp.	0	0	0.3	0	0.8	2.9	0	0	0	2.6
*Psychroserpens* sp.	0.9	1.7	0	0	0.3	1.0	1.4	0	0	0
*Lutibacter* sp.	0	6.1	1.0	0	0.3	1.8	0	0	0	0
*Polaribacter* sp.	0	5.0	0.3	0	1.9	1.8	0	0	0	0
*Uncultured Flavobacteria*	4.1	6.1	0	0.2	0.8	3.7	1.2	0.5	0.2	0
*Croceibacter* sp.	0.2	0	0.8	0.6	0	0.3	1.0	0	1.1	0
*Arenibacter* sp.	2.7	0	0	0	0	0	0	0	0	0
*Maribacter* sp.	0.2	0	0.3	0	0.3	0.5	0	0	0	2.3
*Crocinitomix* sp.	0.2	0.6	0	0	7.1	1.8	0.7	0.2	1.1	2.3
*Owenweeksia* sp.	7.5	0.5	0	0	0	0.8	0	0	0	0
*Balneola sp*.	0.7	0.3	0	0	5.7	0	0	0	0	0
***FIRMICUTES***										
LOHAFEX Cluster 2	0	0	73.9	0	0	0	50.7	0	53.4	0
LOHAFEX Cluster 3	0	0	0	87.7	0	0	0	0	0	0
*Papillibacter* sp.	0	0	0	0	0	0	0	89.8	0	0

#### Dynamics of *Gammaproteobacteria*

Abundance of Gammaproteobacterial clones up to 100 m depth decreased irrespective of whether iron was added (compare abundance at 100 m in St-114 and St-139). Eventually, Gammaproteobacterial clones increased at 500 m depth in both St-114 and St-139 (Figure 3).

At the genera level *Idiomarina sp., Methylophaga* sp., *Alcanivorax* sp., and unclassified Gammaproteobacterial clones appeared at all depths prior to and after fertilization. In addition in iron fertilized water masses *Citrobacter* sp. and *Halomonas* sp. also were detected at all depths. An interesting feature is also the appearance of clones affiliated to genera such as *Thalassolituu*s sp. and *Psychrobacter* sp. at greater depths (beyond 300 m) in the iron fertilized water masses (Table 2) as though these were getting enriched in the presence of iron. *Alcanivorax* sp.and *Psychrobater* sp. showed increase in abundance after iron fertilization (Figure 4B and Table 2). Simultaneously decrease in relative abundance of *Idiomarina* sp., *Citrobacter* sp. *Cycloclasticus* sp. *Methylophaga* sp., and *Marinobacter* sp.was observed (Figure 4B and Table 2).

#### Dynamics of *Bacteroidetes*

Sequences related to *Bacteroidetes* constituted 20.7% of the total clones and the abundance decreased with depth of the water sample (Figure 3). In *Bactereoidetes* clones affiliated to LOHAFEX Cluster1 was the most abundant both prior to and after iron fertilization. It was detectable at all depths prior to fertilization but after iron fertilization they were restricted up to 40 m (Table 2). *Crocinitomix* clones were also detectable at all depths after fertilization. Clones affiliated to *Arenibacter* sp. and *Balneola* sp. were totally absent after iron fertilization whereas clones affiliated to *Lacinutrix* sp. increased in relative abundance following iron fertilization (Figure 4C and Table 2).

#### Dynamics of *Firmicutes*

Clones affiliated to *Firmicutes* were absent in the surface waters and were very much decreased at 500 m (Figure 4D). The total abundance at both the stations was comparable at St-114–300 m and St-139–100 m (Figure 4D).

Clones affiliated to *Firmicutes* are phylogenetically related to LOHAFEX Cluster 2, LOHAFEX Cluster 3, and *Papillibacte*r sp. Clones affiliated to LOHAFEX Cluster 2 and Cluster 3 were detected prior to iron fertilization but the later was not detectable after fertilization (Table 2). In contrast *Papillibacte*r sp. clones appeared only after iron-fertilization. (Figure 4D and Table 2).

The relative abundance based heat map is generated for all the depths before and after fertilization (Figure S3). This also demonstrates that LOHAFEX cluster 2, *Papillibacter* sp., LOHAFEX cluster 3, LOHAFEX cluster 1, *Alcanivorax* sp., and SAR11 cluster were highly anbundant and are distantly clustered to other representatives in all the depths (Figure S3). Based on the heatmap also it is apparent that sequences belonging to *Alcnivorax* sp., and SAR11 cluster are high in number and are present at all depths before and after fertilization. Sequences of *Idiomarina* sp. increased in their number after fertilization and present at all depths. Unclassified *Gammaproteobacterial* and *Methylophaga* sp. were other groups which were present prior to and after fertilization at all depths.

### Mapping of sanger sequences with the 454 reads of Thiele et al. (2012)

**Figure 6:**
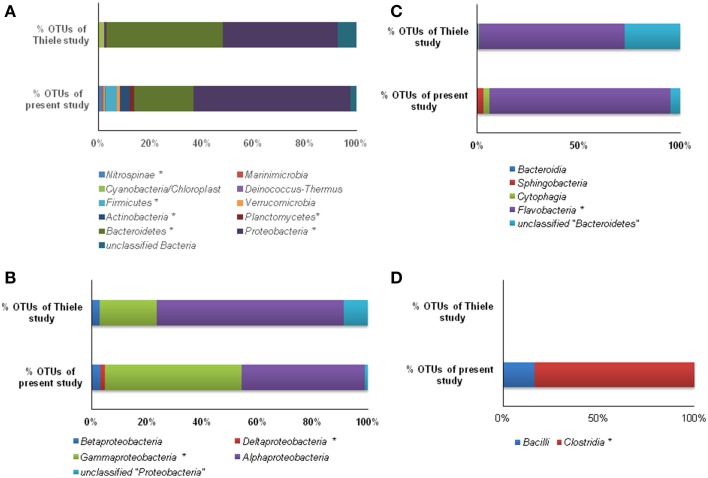
**Mapping of % OTU sequences of present study with the study of Thiele et al. (2012) affiliated to various phyla (A), ***Proteobacteria*** (B), ***Bacteroidetes***, (C) and ***Firmicutes*** (D)**. (^*^, significantly different at 0.01).

Taxonomic distinctions as revealed through hierarchal mapping between Sanger sequences of the present study and 454 read sequences of Thiele et al. (2012) are shown in Table S2. It is clearly evident that the sequences belonging to LOHAFEX cluster 2 and cluster 3 of the present study are not present in 454 read sequences. Sequences of LOHAFEX cluster 1 along with a few 454 read sequences were assigned to “unclassified *Flavobacteriaceae*” group. But sequences of LOHAFEX cluster 1 at genera level were different when compared to 454 read sequences. Phylogenetic analysis also reveals the uniqueness of LOHAFEX cluster 1. Mapping of the percentage OTU sequences of different phyla and classes disclose the differences between the studies (Figures 6A–D). Phylogenetic mapping was done for 277 OTUs of present study with 241 OTUs of Thiele et al. (2012) which includes sequences assigned to “unclassified *Flavobacteriaceae*” group. This revealed that the unique clusters of the present study LOHAFEX cluster 1, cluster 2 and cluster 3 were well separated from the OTUs of Thiele data (Figure S4).

## Discussion

In the current LOHAFEX experiment increase in chlorophyll *a* concentration up to 40 m depth in the iron fertilized water mass is a clear evidence of a phytoplankton bloom (Figure 2A). But this bloom was not as big as the bloom observed in previous iron fertilization experiments namely SOFEX-north and SEED II (Oliver et al., 2004; Boyd et al., 2007; Thiele et al., 2012). The reason could be that these waters were poor in silica. Further, in this study the bloom mainly consisted of *Prymnesiophytes* and not diatoms (Thiele et al., 2012). Increase in total bacterial count in the iron fertilized water mass followed a trend similar to increase in chlorophyll *a* concentration up to 40 m depth (Figure 2B). Simultaneously an increase in leucine incorporation indicative of increase in productivity was also observed (Figure 2D). Surprisingly thymidine incorporation did not increase up to 40 m but was higher at all other depths compared to the unfertilized water mass (Figure 2C). Similar, lack of correlation in thymidine or leucine incorporation compared to bacterial abundance has been also observed previously (Carlson and Ducklow, 1996). Arrieta et al. (2004) demonstrated that in the Southern Ocean leucine and thymidine incorporation in the iron-fertilized patch peaked a few days after iron additions but rapidly decreased to the background levels despite the increase in bacterial abundance.

In LOHAFEX increase in total bacterial count was between 1.1 and 1.9 fold which is lower in comparison to phytoplankton growth (>3 fold) suggesting a weak coupling between bacterioplankton and phytoplankton in the Southern ocean. Grazing by protists (Coale et al., 1996), viral lysis (Cochlan et al., 1993) and availability of dissolved organic matter (Kirchman and Rich, 1997) are the main factors which control the coupling between bacterioplankton and phytoplankton (Ducklow, 1984). Further, high abundance of fecal pellets in the fertilized water mass is also indicative of high grazing pressure (Mazzocchi et al., 2009). Preliminary analysis indicated very high abundance of the copepods *Calanus simillimus, Ctenocalanus citer*, and *Oithona similis*. Copepods are known to graze on prey larger than bacteria (generally protozoans and phytoplankton) while bacteria are generally controlled by protozoans (especially nanoflagellates). Thus in the present study the bacterioplankton appears to be predominantly bottom-up controlled by availability of resources (from phytoplankton or grazing related activities).

The aim of the present study was to confirm that iron fertilization leads to phytoplankton bloom and further to understand how the bloom influences bacterial community structure at different depths through analysis of 16S rRNA gene libraries and phylogenetic analysis. Very high Good's coverage (mean = 94.11%) indicated that the libraries were sampled enough to draw a reasonable conclusion on the abundance and changes in bacterioplankton during the peak of phytoplankton bloom (Table 1). In the 10 clone libraries, sequences related to *Alphaproteobacteria, Gammaproteobacteria, CFB* and *Firmicutes* were dominant and this is in accordance with the previous research on bacterial communities from the Southern ocean (Topping et al., 2006; Jamieson et al., 2012). *Firmicutes* which were not detected in iron fertilization experiments earlier (Arrieta et al., 2004; Kataoka et al., 2009; Obernosterer et al., 2011) is indeed a prominent component of the bacterial community suggesting a functional role for *Firmicutes* in Southern Ocean. We are aware of the fact that the present study would have benefited by replication of sites but this was not possible due to logistic reasons. Nevertheless, the observed Good's coverage and the accompanied distinct changes in the microbial community imply changes following iron fertilization. The present study based on 16S rRNA gene libraries is very highly informative compared to DGGE, t-RFLP and pyro-tag sequencing which were used to analyse the effect of iron fertilization on bacterial community composition (BCC). These results were not consistent since in Eisen Ex, using t-RFLP analysis, BCC was observed to be stable (Arrieta et al., 2004), but in SEEDS-II stimulation in growth of *Roseobacter* clade was observed after iron fertilization (Kataoka et al., 2009).

In a recent study, Thiele et al. (2012) as part of the Lohafex experiment, monitored *bacteria* and *Archaea* over a period of 38 days following iron fertilization. But, the experiment of Thiele et al. (2012) and the present study cannot be strictly compared since the emphasis of the former study was to monitor changes after iron fertilization on day −1, 5, 9, 14, 18, 22, 27, and 36 (IN stations) at 5, 10, 20, and 50 m for bacterial number and bacterial production whereas in the present study the approach was to monitor changes on day -1 and day 14 after iron fertilization but at different depths (0, 40, 100, 300, and 500 m). Thus, we compared our results at 40 m depth on day 14 with that of the data of Thiele et al. (2012) and observed that the two studies showed similar increase in leucine uptake, increase in thymidine uptake (only after 40 m depth) and only slight increase in total cell numbers. In addition in the present study all the three parameters showed increase at all depths below 40 m implying that iron fertilization increased bacterial production and abundance. Further, the 454 pyrotag sequences of Thiele study (Thiele et al., 2012) and 16S rDNA sequences of the present study were differentiated through mapping the sequences. For this purpose, both the datasets were processed using “MOTHUR” software and OTUs were assigned at a threshold limit of 97% similarity. Then the sequences within these two data sets were compared and mapped using Ribosomal database project library compare web tool (https://rdp.cme.msu.edu/comparison/comp.jsp). Mapping results indicated that OTUs (%) in six phyla viz., *Nitrospinae, Firmicutes, Actinobacteria, Planctomycetes, Bacteroidetes*, and *Proteobacteria* were significantly different (Figure 6A). Three of these phyla that were predominantly present and significantly differed viz., *Proteobacteria, Bacteroidetes*, and *Firmicutes* were further mapped at the class level (Figures 6B–D). Hierarchal mapping of all the OTUs of both the studies is presented in Table S2.

Phylogenetic analysis of representative sequences of OTUs of both the studies indicated that compared to Thiele's study, the present study at a time point 14 days following iron fertilization revealed previously unseen diversity along with unique Lohafex clusters I, II, and III (Figure 5 and Table S2). The discrepancy in the results of the present study with that of Thiele et al. (2012) could be attributed to the variation in the approaches with 454 pyro-taging providing smaller length of sequences for phylogenetic analysis (50 to ~450 bp) whereas our study yielded about ~1500 bp length of 16S rRNA gene sequence which is more reliable for phylogenetic analysis. In addition, primer bias could also be a possible reason for the discrepancy. But, this is less likely because Thiele et al. (2012) have generated sequences of the V3-V4 region of 16S rRNA gene and used these sequences for phylogenetic analysis which is widely accepted.

The number of taxa representing different bacterial groups viz., *Alphaproteobacteria, Gammaproteobacteria, Bacteroidetes*, and *Firmicutes* varied in the present study when compared to the 454 pyro-taging study by Thiele et al. (2012). In addition several clone sequences affiliated to *Deinococcus-Thermus*, and *Nitrospinae* were also detected in the present study and not in the study by Thiele et al. (2012). he present study and that of Thiele et al. (2012) indicated a decrease in bacterial cell numbers with depth and more prominent decrease occurring after 40 m depth toward 500 m (Figure 2B). Mapping of the sequence data indicated that *Alphaproteobacteria, Betaproteobacteria*, and *Gammaproteobacteria* are commonly observed in both datasets whereas sequences belonging to *Deltaproteobacteria* were observed only in the present study (Figure 6B and Table S2). Among *Alphaproteobacteria*, as observed by Thiele et al. (2012), in the present study also *Roseobacter* clade, SAR11 clade, SAR116 clade, *Candidatus* Pelagibacter and *Rhodobacteriaceae* members were detected. The present study also demonstrated that within the *Roseobacter* clade, genera comprising *Octadecabacter, Roseovarius, and Sulfitobacter* were predominant and they responded to Iron fertilization. Interestingly within this clade, *Sulfitobacter* spp. increased in their number after fertilization while *Octadecabacter* spp. decreased in their number. Sequence mapping of data sets of both studies indicate that taxa belonging to *Rhodobacteriales, Rhizobiales, Sphingomonadales*, and *Rhodospirillales* showed significant differences (Table S2). The present study also identifies species belonging to *Hyphomonadaceae, Aurantimonadaceae*, and *Phyllobacteriaceae* belonging to *Alphaproteobacteria* (Table S2). After iron fertilization *Gammaproteobacteria* decreased up to 100 m depth and then increased subsequently up to 500 m depth (Figure 3). This is in contrast to the findings of Thiele et al. (2012) and Hutchins et al. (2001a) who observed that *Gammaproteobacteria* showed either no response or only a minor response to the bloom in the sub-antarctic Southern Ocean. But in HNLC waters of the subarctic Pacific and the California coastal upwelling region the responsive phylotypes were related to *Gammaproteobacteria* (Hutchins et al., 2001b). In natural iron fertilization also, *Gammaproteobacteria* (SAR92) were dominant contributors to abundance (Sugita et al., 2012). At the genera level the total abundance calculated for all the depths indicated that *Idiomarina* sp., *Citrobacter* sp. *Cycloclasticus* sp. *Methylophaga* sp., *Marinobacter* sp, and *Pseudomonas* sp. decreased whereas *Halomonas* sp., *Alcanivorax* sp. and *Psychrobacter* sp. increased in the iron fertilized water masses (Table 2) compared to the unfertilized water as though these genera were getting enriched in the presence of iron. Increase in *Psychorbacter* in the surface waters following iron fertilization is surprising since these bacteria are known to produce siderophores (Malmstrom et al., 2004) in iron limiting conditions. So increase in the abundance might be related to the algal bloom scavenging rather than iron scavenging. Thiele et al. (2012) indicated that clones affiliated to class *Gammaproteobacteria* and order *Alteromonadales* decreased following fertilization. Further, the hierarchal maping of the sequences of both studies showed significant differences among sequences belonging to *Alteromonadales, Oceanospirillales, Thiotrichales*, and *Gammaproteobacteria* incertae sedis (Table S2). The present study confirms that *Alteromonadales* decreased at all depths following iron fertilization and the clones identified with *Idiomarina* sp. and *Marinobacter* sp. Our observations also confirm that the SAR11 clade within the *Alphaproteobacteria* were dominant and decreased at all depths after iron-fertilization (Table 2) as observed earlier by Thiele et al. (2012). It was also demonstrated that many of the clones affiliated to SAR11 cluster (210 clones) were phylogenetically identical (100% similarity) with Candidatus *Pelagibacter ubique* and the others were closely related to uncultured marine bacterial clones from Arctic and Antarctic sea water (Figure S1A and Figure 4A). In addition, in this study it was also observed that SAR116 cluster which is closely related to Candidatus *Puniceispirillum marinum* the first cultured representative of the SAR116 clade decreased with fertilization (Figure 4A and Table 2). Earlier studies have clearly indicated that SAR11, SAR83, and SAR116 clusters of the alpha subclass, and the SAR86 cluster of the gamma subclass are recovered most frequently from marine environmental clone libraries (Giovannoni et al., 1996; Malmstrom et al., 2004, 2005).

*Bacteroidetes* are known to be highly abundant in the Southern Ocean (Alderkamp et al., 2006) and are known to utilize polysaccharides (Gómez-Pereira et al., 2012) and proteins (Pinhassi et al., 1999; Cottrell and Kirchman, 2000). In the present study a greater degree of diversity was observed within the *Bacteroidetes* which was represented by 11 different genera and LOHAFEX Cluster1 (Table 2 and Figure S1C). Clones in LOHAFEX Cluster 1 formed a monophyletic clade and are affiliated to Flavobacterial clones (Figure 5). which have been linked with phytoplankton bloom and degradation of organic matter (Kirchman, 2002; Junge et al., 2004). The nearest clade which is phylogenetically related to LOHAFEX Cluster 1 included species of *Lacinutrix, Psychroserpens* and *Winogradskyella* which were also detected in the present study (Figure S1C). Overall it appears that *Bacteroidetes* decreased after iron fertilization and this is in accordance with the LOHAFEX study of Thiele et al. (2012). Mapping of the sequences of present study with the sequences of Thiele study to find the resemblances for phyla *Bacteroidetes* reveals that sequences belonging to class *Flavobacteria* were predominantly present (Figure 6C). However the hierarchal mapping of the sequences shows that the LOHAFEX Cluster 1 of the present study is rooted to the unclassified *Flavobacteriaceae* (Table S2). Similarly a number of sequences of Thiele study were also assigned as unclassified *Flavobacteriaceae*. Thus, phylogenetic mapping of the sequences of both data sets was done to reveal the relatedness. Phylogenetic sequence mapping of OTUs of Thiele study (labeled as THOTU) with present study (labeled as OUT) demonstrated that LOHAFEX Cluster 1 shows a clear separation from THOTUs belonging to phylum *Bacteroidetes* (Figure S4). Following iron fertilization many of the *Bacteroidetes* genera decreased significantly or were no longer detectable like clones affiliated to *Arenibacter sp. Owenweeksia sp*. and *Balneola sp*. (Figure 4C). Genera *Lacinutri* and, *Croceibacter* significantly increased in the iron fertilized water masses implicating their role in degradation of the bloom (Figure 4C). In the present study majority of the sequences of phyla *Bacteroidetes* belonged to LOHAFEX cluster 1 (Flavobacteria).

Sequences belonging to *Firmicutes* of the present study were clearly distinct from sequences of Thiele's study as revealed by hierarchy mapping of both datasets (Figure 6D and Table S2). *Firmicutes* represented by LOHAFEX Cluster 2, and LOHAFEX Cluster 3 responded to the iron-induced algal bloom in three different ways. LOHAFEX Cluster 2 increased after iron-fertilization and LOHAFEX Cluster 3 disappeared after iron- fertilization (Figure 4D). Clones of both these clusters are monophyletic and are phylogenetically related to genera *Papillibacter* and *Clostridium* (Figure 5) which are common in marine waters. Cultivable species of *Clostridium* and uncultured environmental clones (4Bac R8 and Vadin HA31) are known to be involved in biodegradation (Bengelsdorf et al., 2013). LOHAFEX Cluster 2 is also closely related to a clade of *Papillibacter cinnamivorans* involved in cinnamate transformation (Defnoun et al., 2000), which in the present study is represented by a single OTU218 with 388 clones only in the iron fertilized water (Figure 5) clearly implying a role for this species in degradation. The preponderance and appearance of OTU218 only in iron fertilized waters may imply that *Papillibacter* sp. is an indicator species of iron-fertilized waters. Clones affiliated to *Firmicutes* have been detected between 40 and 300 m depth in 5 out of the 10 libraries (Table 2) though this has not been reported in earlier studies. In a recent study *Firmicutes* were detected in multiple sites of Scotia Sea and abundance ranged from 9 to 15 × 10^−3^-cells ml^−1^(Topping et al., 2006). In the study of Thiele et al. (2012) clones affiliated to *Firmicutes* was very minimal (Figure 6C and Table S2).

In addition a number of other genera like Sulfitobacter sp., Thalassospira sp., Sphingopyxis sp., Lacinutrix sp., Crocinitomix sp., Alcanivorax sp., showed increase where as others like Lutibacter sp., Polaribacter sp., Arenibacter sp., Owenweeksia sp., and Balneola sp. etc. showed a decrease in abundance following iron fertilization thus confirming a succession like response upon the algal bloom (Figures 4A–D).

Alpha diversity mainly focuses on the number and abundance of the species while beta is mainly related to the phylogenetic structure of the community. In this study statistical analysis indicated a decrease in alpha diversity in fertilized water column as evidenced by ChaoI and H', except 300 m depth where there is an increase in H' after iron fertilization. The decrease in the alpha diversity indices after iron fertilization also supports the bottom up control on bacteria community composition. Results, from Unifrac analysis confirmed a decrease in bacterial community diversity but the phylogenetic structure of the community was unchanged. Our hypothesis is that the observed decrease in bacterial community diversity in the bloom is due to limitation in the supply of nutrients which are in increased demand due to increase in biomass thus causing a reduction in number of some species. But this reduction did not affect the overall bacterial community phylogenetic structure.

Thus, the present study confirms that following iron fertilization in the Southern Ocean a phytoplankton bloom occurs (Boyd et al., 2007; Thiele et al., 2012), bacterial abundance and production increased (Blain et al., 2007; Obernosterer et al., 2007) and the bacterial communities changed following iron fertilization and with depth of water (Figures 3, 4 and Table 2). Earlier studies did not exhibit significant differences prior to and after iron fertilization in the bacterial communities (Hutchins et al., 2001a,b; Arrieta et al., 2004; Blain et al., 2007). *Alphaproteobacteria, Gammaproteobacteria*, and *Bacteroidetes* were the dominant clones as observed earlier (Pakulski et al., 1996; Cochlan, 2001) but in addition clones affiliated to *Firmicutes* were also consistently detected. Specific genera in each of the above phyla increased, decreased or appeared following iron fertilization implying a dynamic change in the bacterial community following iron fertilization and algal bloom. For instance, *Roseobacter* clade, SAR11 and SAR116 clades within the *Alphaproteobacteria*, are dominant and decreased at all depths after iron-fertilization. A greater degree of diversity was observed within the *Bacteroidetes* represented by11 different genera and LOHAFEX Cluster 1.

Overall it appears that *Bacteroidetes* decreased after iron fertilization though a few genera like *Lacinutrix, Croceibacter* and *Crocinitomix* increased in the iron fertilized water masses implicating their role in degradation of the bloom. *Firmicutes* represented by LOHAFEX Cluster 2, LOHAFEX Cluster 3 and *Papillibacte*r sp. also responded to the iron fertilization induced algal bloom as indicated by the appearance of *Papillibacter* sp., increase in LOHAFEX Cluster 2 and disappearance of LOHAFEX Cluster 3 after iron fertilization. The phylogenetic clusters LOHAFEX Cluster 1, 2, and 3 were not detected in any of the earlier studies and it is implied that clones affiliated to *Firmicutes* in addition to those affiliated to *Alphaproteobacteria, Gammaproteobacteria* and *Bacteroidetes* play an important role in scavenging of phytoplankton blooms induced following iron fertilization. It is hypothesized that heterotrophic bacteria could utilize algal exudates and decaying algal biomass thus resulting in a dynamic bacterial succession of distinct genera. It is interesting to note that the short sequence reads from the 454 pyro tagging approach that covered V3-V4 region (341F/805R) of 16S rRNA gene could not identify the LOHAFEX clusters while the present study based on 16S rDNA clone library approach did. On the whole the sequence mapping of 454 sequence reads with 16S rDNA sequence clone libraries clearly show that the later method still holds its “golden standard” for metagenomic analysis. Though both have their advantages and disadvantges, the clone library method appears to be more persuasive due to the maximum coverage of the 16S rRNA gene length which is done by using multiple primers (27F/1489R) for the analysis.

### Conflict of interest statement

The authors declare that the research was conducted in the absence of any commercial or financial relationships that could be construed as a potential conflict of interest.

## Supplementary material

Figure S1 **Neighbor joining phylogenetic tree of 16S rRNA gene clones from all 277 OTUs of ten libraries of stations 139 and 114, showing the phylogenetic relationship of clones affiliated to ***Alphaproteobacteria*** (A), ***Gammaproteobacteria*** (B), ***Bactereoidetes*** (C), ***Firmicutes*** (D), and Other Bacteria (E)**. Other Bacteria include *Betaproteobacteria*, *Deltaproteobacteria*, cyanobacteria/plastid sequence, *Actinobacteria, Nitrospinae*, *Planctomycetes*, and *Verrucomicrobia*, and *Deinococcus-Thermus*. *Thiohalobacter thiocyanaticus* strain HRh1 (FJ482231) was taken as an out-group for *Alphaproteobacteria* and *Agrobacterium larrymoorei* 3-10^T^ (Z30542) was taken as an out-group for *Gammaproteobacteria*, *Bactereoidetes Firmicutes* and Other bacteria. Numbers at nodes are bootstrap values. The bar represents 0.05 substitutions per alignment position.

Figure S2 **Rarefaction curves of the observed OTUs of the two stations 114 and 139 at different depths**.

Figure S3 **Heat map depicting the comparison of different genera affiliated to ***Alphaproteobacteria***, ***Gammaproteobacteria***, ***Bacteroidetes*** and ***Firmicutes*** in stations St-114 and St-139 at different depths**.

Figure S4 **Phylogenetic mapping of OTUs of present study with Thiele's study (Thiele et al., 2012)**. OUT = OTUs of present study; THOTU = OTUs of Thiele's study.

Table S1 **List of OTUs, OTU representatives and the clone numbers**.

Table S2 **Hierarchy mapping of total OTUs of present study with Thiele data (Thiele et al., 2012)**. Significantly different at 0.01 is highlighted as * /. Parenthesis comprises OTUs of present study/Thiele study/significance value respectively.
