# Supplementary figures and images for: Response of bacterioplankton to iron fertilization of the Southern Ocean, Antarctica
Source: Front Microbiol. 2015 Aug 26;6:863. doi: 10.3389/fmicb.2015.00863 (PMC4550105; doi:10.3389/fmicb.2015.00863)

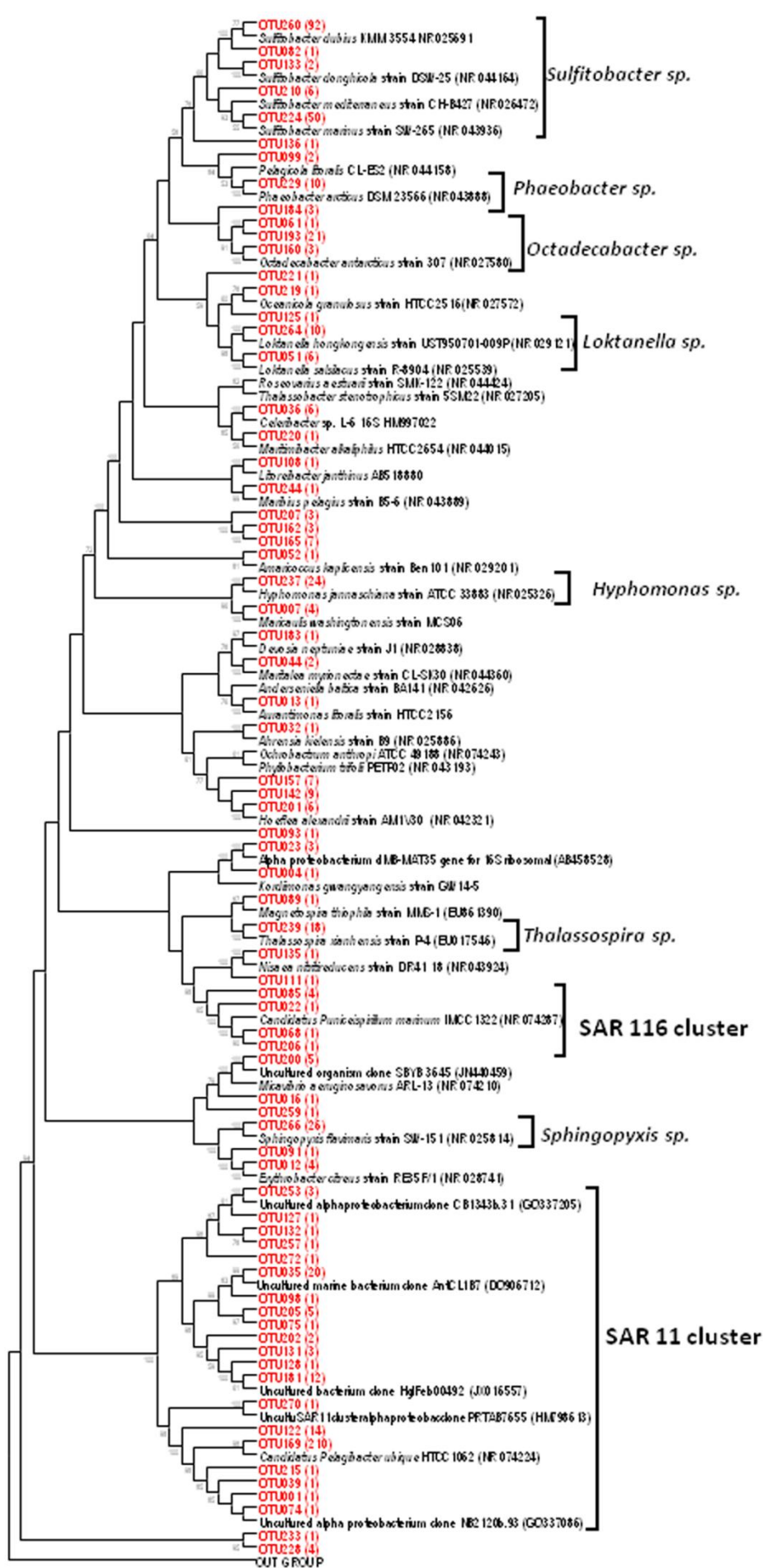

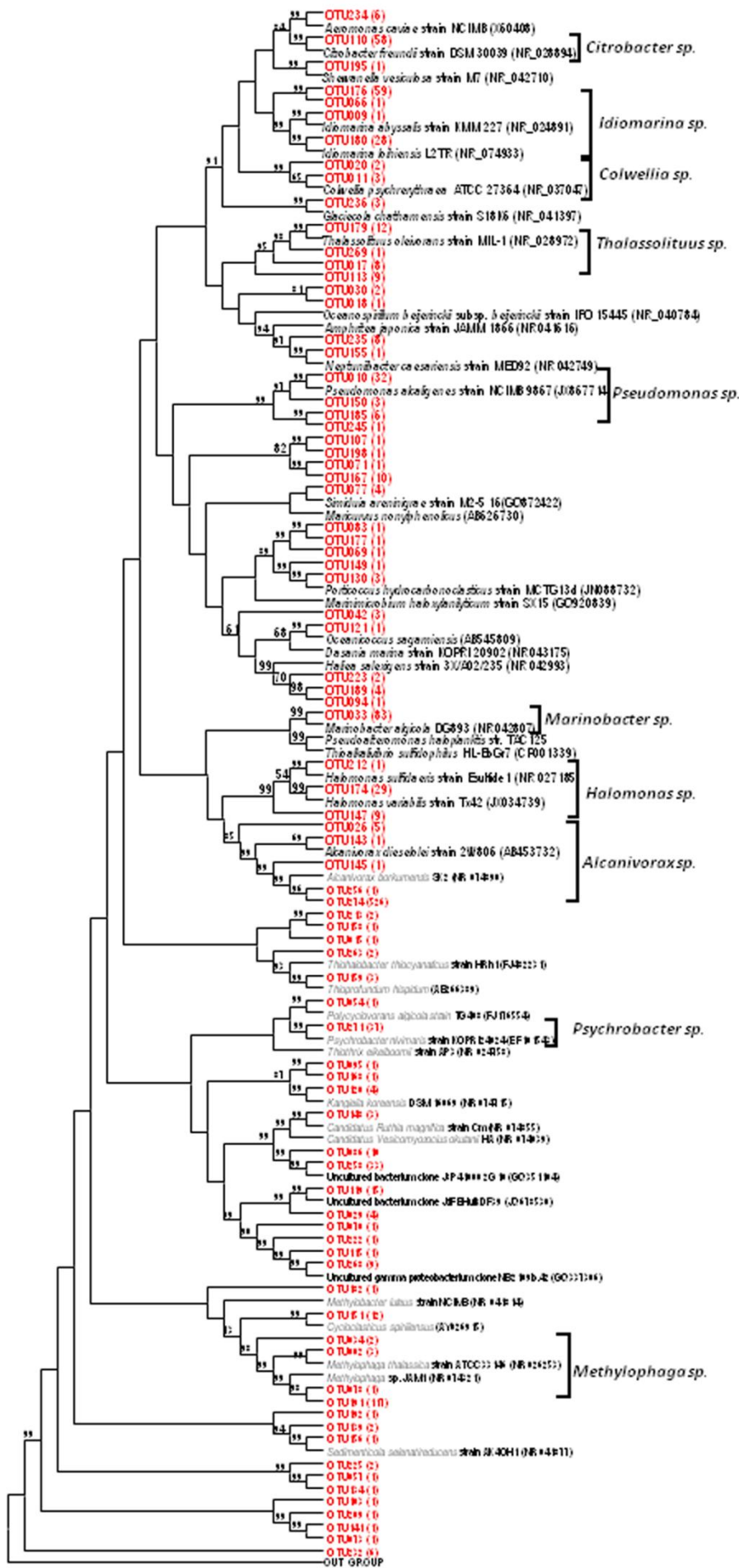

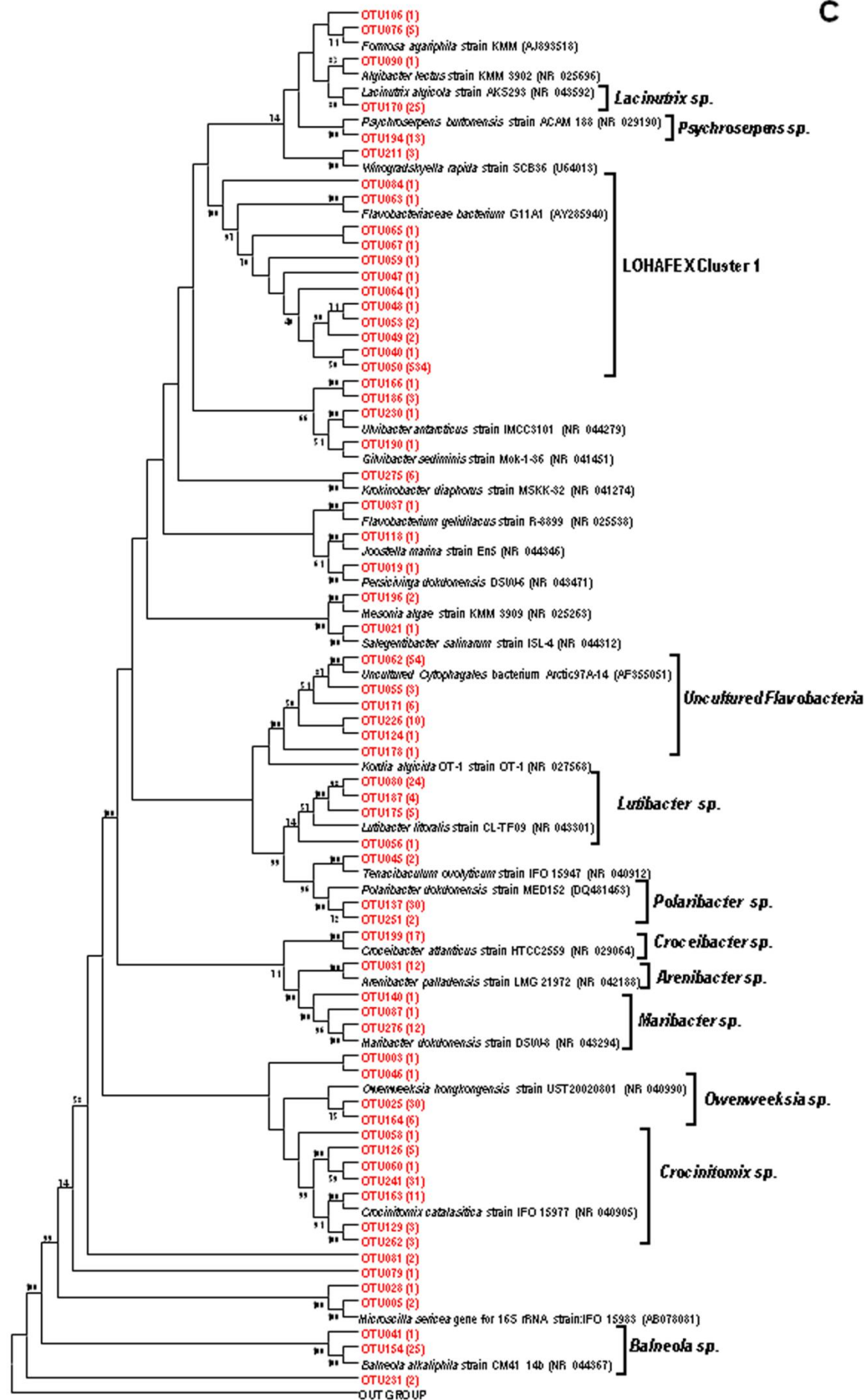

**D**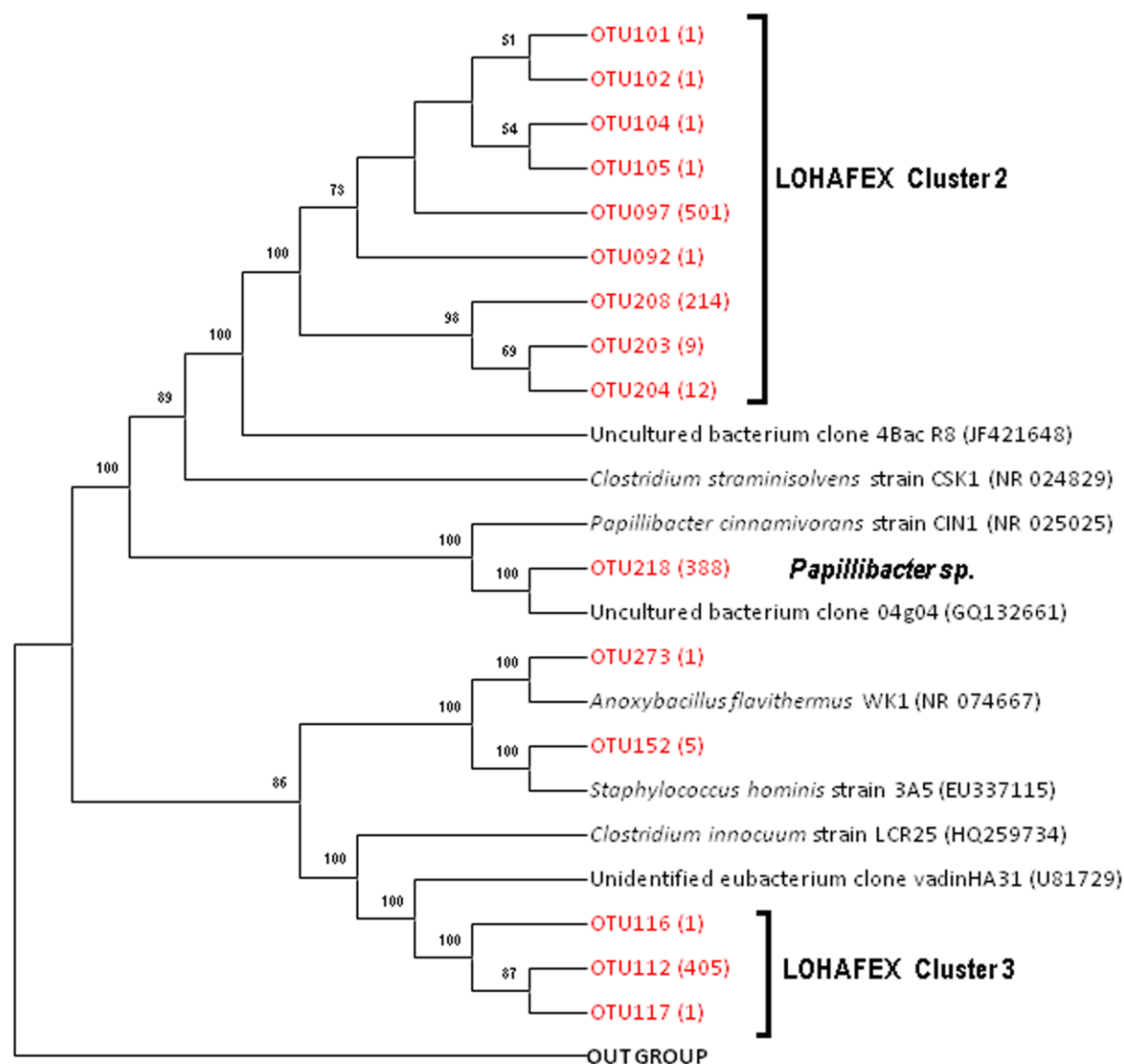

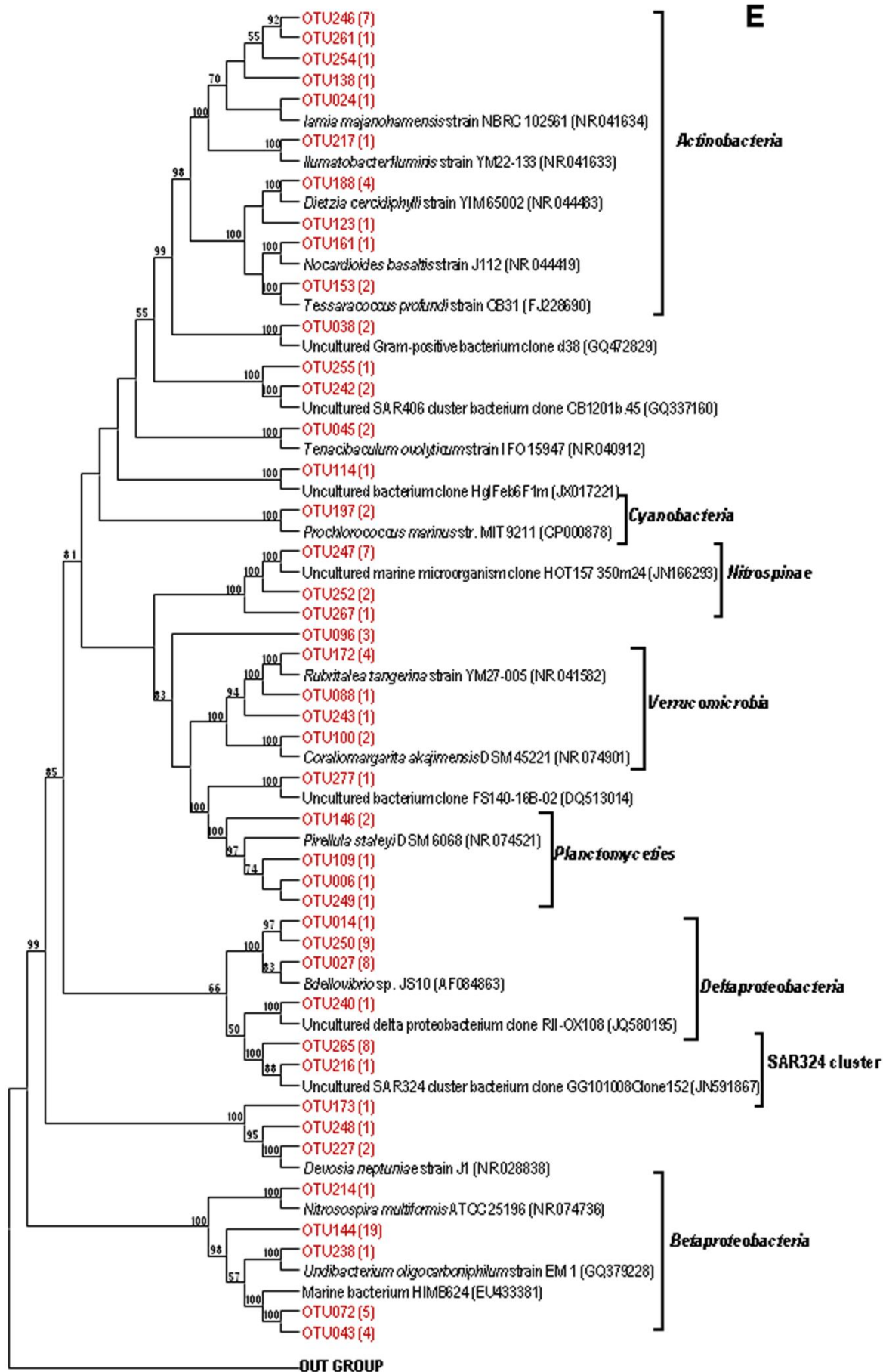

Supplement: Figure S1 — Neighbor joining phylogenetic tree of 16S rRNA gene clones from all 277 OTUs of ten libraries of stations 139 and 114, showing the phylogenetic relationship of clones affiliated to Alphaproteobacteria (A), Gammaproteobacteria (B), Bactereoidetes (C), Firmicutes (D), and Other Bacteria (E). Other Bacteria include Betaproteobacteria, Deltaproteobacteria, cyanobacteria/plastid sequence, Actinobacteria, Nitrospinae, Planctomycetes, and Verrucomicrobia, and Deinococcus-Thermus. Thiohalobacter thiocyanaticus strain HRh1 (FJ482231) was taken as an out-group for Alphaproteobacteria and Agrobacterium larrymoorei 3-10T (Z30542) was taken as an out-group for Gammaproteobacteria, Bactereoidetes Firmicutes and Other bacteria. Numbers at nodes are bootstrap values. The bar represents 0.05 substitutions per alignment position. [file Image1.PDF]

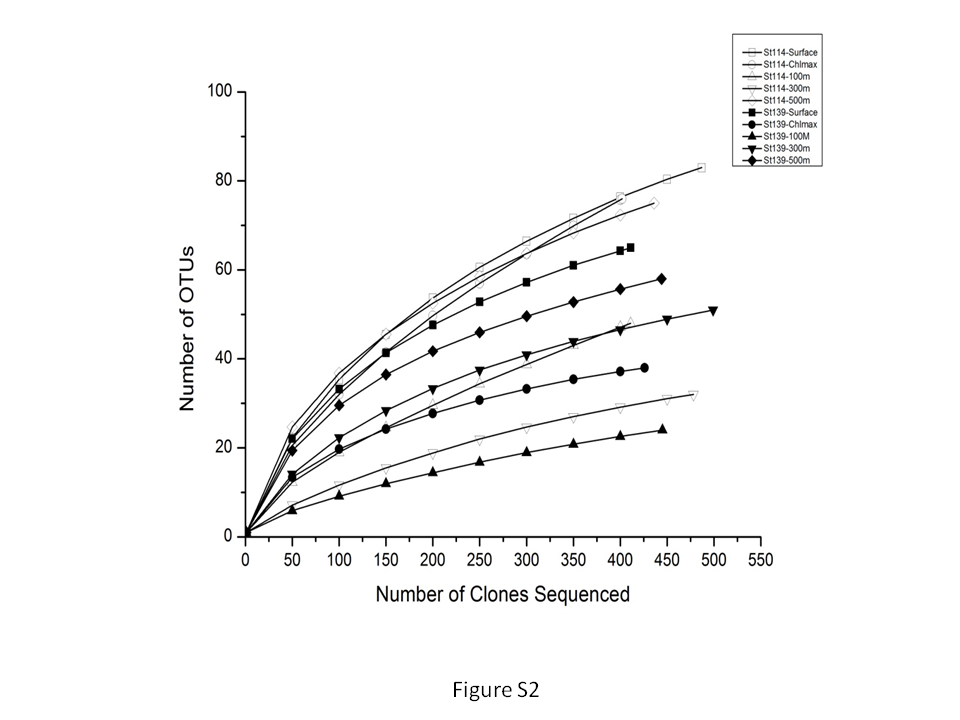

Supplement: Figure S2 — Rarefaction curves of the observed OTUs of the two stations 114 and 139 at different depths. [file Image2.TIF]

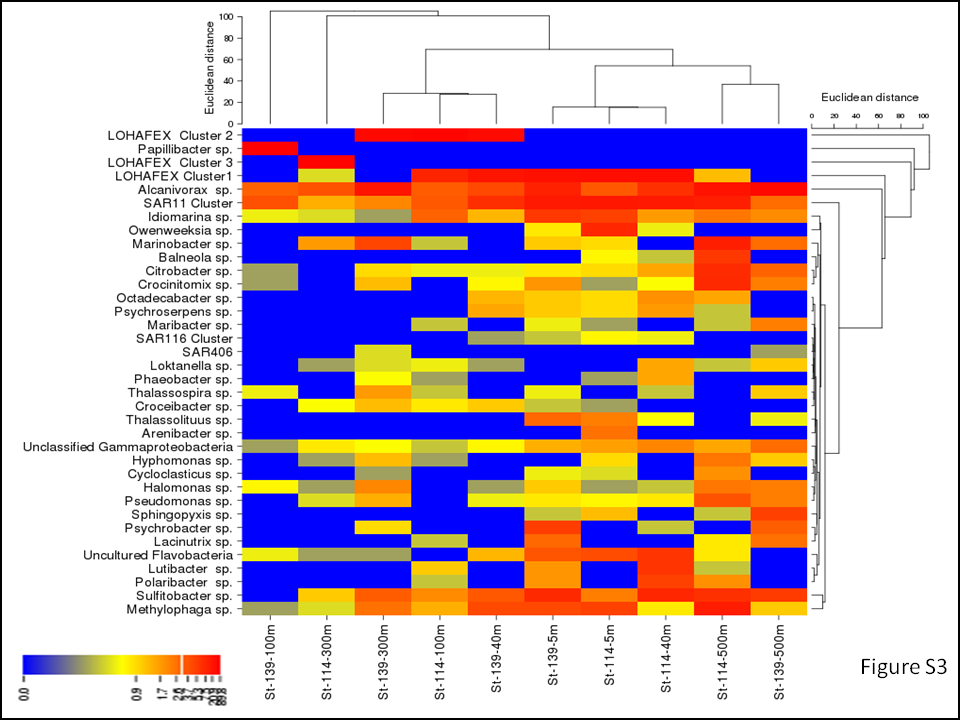

Supplement: Figure S3 — Heat map depicting the comparison of different genera affiliated to Alphaproteobacteria, Gammaproteobacteria, Bacteroidetes and Firmicutes in stations St-114 and St-139 at different depths. [file Image3.TIF]

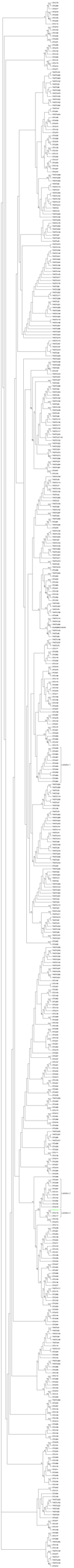

Supplement: Figure S4 — Phylogenetic mapping of OTUs of present study with Thiele's study (Thiele et al., 2012). OUT = OTUs of present study; THOTU = OTUs of Thiele's study. [file Image4.PDF]
