# Supplementary material for: Response of bacterioplankton to iron fertilization of the Southern Ocean, Antarctica
Source: Front Microbiol. 2015 Aug 26;6:863. doi: 10.3389/fmicb.2015.00863 (PMC4550105; doi:10.3389/fmicb.2015.00863)
Supplement: Table S2 — Hierarchy mapping of total OTUs of present study with Thiele data (Thiele et al., 2012). Significantly different at 0.01 is highlighted as * /. Parenthesis comprises OTUs of present study/Thiele study/significance value respectively. [file Table2.DOCX]

**Table S2. Hierarchy mapping of total OTUs of present study with Thiele data (Thiele et al., 2012).** Parenthesis comprises OTUs of Presents study/Thielestudy/significance value respectively. Significantly different at 0.01 is highlighted as * /  .

| »  »  »  phylum [Nitrospinae](https://rdp.cme.msu.edu/comparison/comp_hierarchy.jsp?root=2756&depth=10&confidence=0.95) (3/0/5.44E-4)  »  »  »  »  class [Nitrospinia](https://rdp.cme.msu.edu/comparison/comp_hierarchy.jsp?root=2757&depth=10&confidence=0.95) (3/0/5.44E-4)  »  »  »  »  »  order [Nitrospinales](https://rdp.cme.msu.edu/comparison/comp_hierarchy.jsp?root=2758&depth=10&confidence=0.95) (3/0/5.44E-4)  »  »  »  »  »  »  family [Nitrospinaceae](https://rdp.cme.msu.edu/comparison/comp_hierarchy.jsp?root=1393&depth=10&confidence=0.95) (3/0/5.44E-4)  »  »  »  »  »  »  »  genus [Nitrospina](https://rdp.cme.msu.edu/comparison/comp_hierarchy.jsp?root=2961&depth=10&confidence=0.95) (3/0/5.44E-4) |
| --- |
| »  »  »  phylum [Marinimicrobia](https://rdp.cme.msu.edu/comparison/comp_hierarchy.jsp?root=2728&depth=10&confidence=0.95) (2/2/3.46E-2)  »  »  »  »  genus [Marinimicrobia_genera_incertae_sedis](https://rdp.cme.msu.edu/comparison/comp_hierarchy.jsp?root=2729&depth=10&confidence=0.95) (2/2/3.46E-2) |
| »  »  »  phylum ["Verrucomicrobia"](https://rdp.cme.msu.edu/comparison/comp_hierarchy.jsp?root=2164&depth=10&confidence=0.95) (4/5/1E-2)  »  »  »  »  class [Opitutae](https://rdp.cme.msu.edu/comparison/comp_hierarchy.jsp?root=2165&depth=10&confidence=0.95) (1/0/3.3E-2)  »  »  »  »  »  order [Puniceicoccales](https://rdp.cme.msu.edu/comparison/comp_hierarchy.jsp?root=2170&depth=10&confidence=0.95) (1/0/3.3E-2)  »  »  »  »  »  »  family [Puniceicoccaceae](https://rdp.cme.msu.edu/comparison/comp_hierarchy.jsp?root=2171&depth=10&confidence=0.95) (1/0/3.3E-2)  »  »  »  »  »  »  »  genus [Coraliomargarita](https://rdp.cme.msu.edu/comparison/comp_hierarchy.jsp?root=2173&depth=10&confidence=0.95) (1/0/3.3E-2)  »  »  »  »  class [Verrucomicrobiae](https://rdp.cme.msu.edu/comparison/comp_hierarchy.jsp?root=2183&depth=10&confidence=0.95) (2/5/1.44E-1)  »  »  »  »  »  order [Verrucomicrobiales](https://rdp.cme.msu.edu/comparison/comp_hierarchy.jsp?root=2184&depth=10&confidence=0.95) (2/5/1.44E-1)  »  »  »  »  »  »  family ["Rubritaleaceae"](https://rdp.cme.msu.edu/comparison/comp_hierarchy.jsp?root=2185&depth=10&confidence=0.95) (1/1/9.05E-2)  »  »  »  »  »  »  »  genus [Rubritalea](https://rdp.cme.msu.edu/comparison/comp_hierarchy.jsp?root=2186&depth=10&confidence=0.95) (1/1/9.05E-2)  »  »  »  »  »  »  family [Verrucomicrobiaceae](https://rdp.cme.msu.edu/comparison/comp_hierarchy.jsp?root=2187&depth=10&confidence=0.95) (1/3/2.53E-1)  »  »  »  »  »  »  »  genus [Persicirhabdus](https://rdp.cme.msu.edu/comparison/comp_hierarchy.jsp?root=2191&depth=10&confidence=0.95) (0/1/1.52E0)  »  »  »  »  »  »  »  [unclassified Verrucomicrobiaceae](https://rdp.cme.msu.edu/comparison/comp_hierarchy.jsp?root=-2187&depth=10&confidence=0.95) (1/2/NA)  »  »  »  »  »  »  [unclassified Verrucomicrobiales](https://rdp.cme.msu.edu/comparison/comp_hierarchy.jsp?root=-2184&depth=10&confidence=0.95) (0/1/NA)  »  »  »  »  [unclassified "Verrucomicrobia"](https://rdp.cme.msu.edu/comparison/comp_hierarchy.jsp?root=-2164&depth=10&confidence=0.95) (1/0/NA) |
| »  »  »  phylum [Firmicutes](https://rdp.cme.msu.edu/comparison/comp_hierarchy.jsp?root=2259&depth=10&confidence=0.95) (12/1/6.37E-11)  »  »  »  »  class [Bacilli](https://rdp.cme.msu.edu/comparison/comp_hierarchy.jsp?root=2260&depth=10&confidence=0.95) (2/1/1.53E-2)  »  »  »  »  »  order [Lactobacillales](https://rdp.cme.msu.edu/comparison/comp_hierarchy.jsp?root=2333&depth=10&confidence=0.95) (0/1/1.52E0)  »  »  »  »  »  »  family [Carnobacteriaceae](https://rdp.cme.msu.edu/comparison/comp_hierarchy.jsp?root=2342&depth=10&confidence=0.95) (0/1/1.52E0)  »  »  »  »  »  »  »  genus [Carnobacterium](https://rdp.cme.msu.edu/comparison/comp_hierarchy.jsp?root=2354&depth=10&confidence=0.95) (0/1/1.52E0)  »  »  »  »  »  order [Bacillales](https://rdp.cme.msu.edu/comparison/comp_hierarchy.jsp?root=2261&depth=10&confidence=0.95) (2/0/4.24E-3)  »  »  »  »  »  »  family [Bacillaceae 1](https://rdp.cme.msu.edu/comparison/comp_hierarchy.jsp?root=2264&depth=10&confidence=0.95) (1/0/3.3E-2)  »  »  »  »  »  »  »  genus [Anoxybacillus](https://rdp.cme.msu.edu/comparison/comp_hierarchy.jsp?root=2267&depth=10&confidence=0.95) (1/0/3.3E-2)  »  »  »  »  »  »  family [Staphylococcaceae](https://rdp.cme.msu.edu/comparison/comp_hierarchy.jsp?root=2320&depth=10&confidence=0.95) (1/0/3.3E-2)  »  »  »  »  »  »  »  genus [Staphylococcus](https://rdp.cme.msu.edu/comparison/comp_hierarchy.jsp?root=2322&depth=10&confidence=0.95) (1/0/3.3E-2)  »  »  »  »  class [Clostridia](https://rdp.cme.msu.edu/comparison/comp_hierarchy.jsp?root=2375&depth=10&confidence=0.95) (10/0/3.13E-10)  »  »  »  »  »  order [Clostridiales](https://rdp.cme.msu.edu/comparison/comp_hierarchy.jsp?root=2376&depth=10&confidence=0.95) (10/0/3.13E-10)  »  »  »  »  »  »  family [Ruminococcaceae](https://rdp.cme.msu.edu/comparison/comp_hierarchy.jsp?root=2484&depth=10&confidence=0.95) (10/0/3.13E-10)  »  »  »  »  »  »  »  [unclassified Ruminococcaceae](https://rdp.cme.msu.edu/comparison/comp_hierarchy.jsp?root=-2484&depth=10&confidence=0.95) (10/0/NA) |
| »  »  »  phylum ["Deinococcus-Thermus"](https://rdp.cme.msu.edu/comparison/comp_hierarchy.jsp?root=758&depth=10&confidence=0.95) (1/0/3.3E-2)  »  »  »  »  class [Deinococci](https://rdp.cme.msu.edu/comparison/comp_hierarchy.jsp?root=759&depth=10&confidence=0.95) (1/0/3.3E-2)  »  »  »  »  »  order [Thermales](https://rdp.cme.msu.edu/comparison/comp_hierarchy.jsp?root=767&depth=10&confidence=0.95) (1/0/3.3E-2)  »  »  »  »  »  »  family [Thermaceae](https://rdp.cme.msu.edu/comparison/comp_hierarchy.jsp?root=768&depth=10&confidence=0.95) (1/0/3.3E-2)  »  »  »  »  »  »  »  genus [Thermus](https://rdp.cme.msu.edu/comparison/comp_hierarchy.jsp?root=772&depth=10&confidence=0.95) (1/0/3.3E-2) |
| »  »  »  phylum ["Planctomycetes"](https://rdp.cme.msu.edu/comparison/comp_hierarchy.jsp?root=824&depth=10&confidence=0.95) (5/5/2.32E-3)  »  »  »  »  class [Phycisphaerae](https://rdp.cme.msu.edu/comparison/comp_hierarchy.jsp?root=837&depth=10&confidence=0.95) (1/1/9.05E-2)  »  »  »  »  »  order [Phycisphaerales](https://rdp.cme.msu.edu/comparison/comp_hierarchy.jsp?root=838&depth=10&confidence=0.95) (1/1/9.05E-2)  »  »  »  »  »  »  family [Phycisphaeraceae](https://rdp.cme.msu.edu/comparison/comp_hierarchy.jsp?root=839&depth=10&confidence=0.95) (1/1/9.05E-2)  »  »  »  »  »  »  »  genus [Phycisphaera](https://rdp.cme.msu.edu/comparison/comp_hierarchy.jsp?root=840&depth=10&confidence=0.95) (1/1/9.05E-2)  »  »  »  »  class [Planctomycetia](https://rdp.cme.msu.edu/comparison/comp_hierarchy.jsp?root=825&depth=10&confidence=0.95) (4/4/5.61E-3)  »  »  »  »  »  order [Planctomycetales](https://rdp.cme.msu.edu/comparison/comp_hierarchy.jsp?root=826&depth=10&confidence=0.95) (4/4/5.61E-3)  »  »  »  »  »  »  family [Planctomycetaceae](https://rdp.cme.msu.edu/comparison/comp_hierarchy.jsp?root=827&depth=10&confidence=0.95) (4/4/5.61E-3)  »  »  »  »  »  »  »  genus [Planctomyces](https://rdp.cme.msu.edu/comparison/comp_hierarchy.jsp?root=832&depth=10&confidence=0.95) (1/0/3.3E-2)  »  »  »  »  »  »  »  [unclassified Planctomycetaceae](https://rdp.cme.msu.edu/comparison/comp_hierarchy.jsp?root=-827&depth=10&confidence=0.95) (3/4/NA) |
| »  »  »  phylum ["Bacteroidetes"](https://rdp.cme.msu.edu/comparison/comp_hierarchy.jsp?root=422&depth=10&confidence=0.95) (64/785/3.65E-9)  »  »  »  »  class ["Bacteroidia"](https://rdp.cme.msu.edu/comparison/comp_hierarchy.jsp?root=423&depth=10&confidence=0.95) (0/1/1.52E0)  »  »  »  »  »  order ["Bacteroidales"](https://rdp.cme.msu.edu/comparison/comp_hierarchy.jsp?root=424&depth=10&confidence=0.95) (0/1/1.52E0)  »  »  »  »  »  »  family ["Prevotellaceae"](https://rdp.cme.msu.edu/comparison/comp_hierarchy.jsp?root=448&depth=10&confidence=0.95) (0/1/1.52E0)  »  »  »  »  »  »  »  genus [Prevotella](https://rdp.cme.msu.edu/comparison/comp_hierarchy.jsp?root=451&depth=10&confidence=0.95) (0/1/1.52E0)  »  »  »  »  class [Sphingobacteriia](https://rdp.cme.msu.edu/comparison/comp_hierarchy.jsp?root=565&depth=10&confidence=0.95) (2/1/1.53E-2)  »  »  »  »  »  order ["Sphingobacteriales"](https://rdp.cme.msu.edu/comparison/comp_hierarchy.jsp?root=566&depth=10&confidence=0.95) (2/1/1.53E-2)  »  »  »  »  »  »  family [Sphingobacteriaceae](https://rdp.cme.msu.edu/comparison/comp_hierarchy.jsp?root=648&depth=10&confidence=0.95) (0/1/1.52E0)  »  »  »  »  »  »  »  [unclassified Sphingobacteriaceae](https://rdp.cme.msu.edu/comparison/comp_hierarchy.jsp?root=-648&depth=10&confidence=0.95) (0/1/NA)  »  »  »  »  »  »  family [Chitinophagaceae](https://rdp.cme.msu.edu/comparison/comp_hierarchy.jsp?root=567&depth=10&confidence=0.95) (2/0/4.24E-3)  »  »  »  »  »  »  »  genus [Balneola](https://rdp.cme.msu.edu/comparison/comp_hierarchy.jsp?root=568&depth=10&confidence=0.95) (2/0/4.24E-3)  »  »  »  »  class [Cytophagia](https://rdp.cme.msu.edu/comparison/comp_hierarchy.jsp?root=2767&depth=10&confidence=0.95) (2/1/1.53E-2)  »  »  »  »  »  order [Cytophagales](https://rdp.cme.msu.edu/comparison/comp_hierarchy.jsp?root=2768&depth=10&confidence=0.95) (2/1/1.53E-2)  »  »  »  »  »  »  family [Flammeovirgaceae](https://rdp.cme.msu.edu/comparison/comp_hierarchy.jsp?root=622&depth=10&confidence=0.95) (2/0/4.24E-3)  »  »  »  »  »  »  »  genus [Fulvivirga](https://rdp.cme.msu.edu/comparison/comp_hierarchy.jsp?root=627&depth=10&confidence=0.95) (1/0/3.3E-2)  »  »  »  »  »  »  »  genus [Marivirga](https://rdp.cme.msu.edu/comparison/comp_hierarchy.jsp?root=631&depth=10&confidence=0.95) (1/0/3.3E-2)  »  »  »  »  »  »  [unclassified Cytophagales](https://rdp.cme.msu.edu/comparison/comp_hierarchy.jsp?root=-2768&depth=10&confidence=0.95) (0/1/NA)  »  »  »  »  class [Flavobacteriia](https://rdp.cme.msu.edu/comparison/comp_hierarchy.jsp?root=458&depth=10&confidence=0.95) (57/566/1.08E-3)  »  »  »  »  »  order ["Flavobacteriales"](https://rdp.cme.msu.edu/comparison/comp_hierarchy.jsp?root=459&depth=10&confidence=0.95) (57/566/1.08E-3)  »  »  »  »  »  »  family [Flavobacteriaceae](https://rdp.cme.msu.edu/comparison/comp_hierarchy.jsp?root=470&depth=10&confidence=0.95) (47/426/3.24E-2)  »  »  »  »  »  »  »  genus [Dokdonia](https://rdp.cme.msu.edu/comparison/comp_hierarchy.jsp?root=487&depth=10&confidence=0.95) (1/0/3.3E-2)  »  »  »  »  »  »  »  genus [Winogradskyella](https://rdp.cme.msu.edu/comparison/comp_hierarchy.jsp?root=559&depth=10&confidence=0.95) (1/0/3.3E-2)  »  »  »  »  »  »  »  genus [Croceibacter](https://rdp.cme.msu.edu/comparison/comp_hierarchy.jsp?root=485&depth=10&confidence=0.95) (1/0/3.3E-2)  »  »  »  »  »  »  »  genus [Mesonia](https://rdp.cme.msu.edu/comparison/comp_hierarchy.jsp?root=526&depth=10&confidence=0.95) (1/0/3.3E-2)  »  »  »  »  »  »  »  genus [Psychroserpens](https://rdp.cme.msu.edu/comparison/comp_hierarchy.jsp?root=539&depth=10&confidence=0.95) (1/0/3.3E-2)  »  »  »  »  »  »  »  genus [Joostella](https://rdp.cme.msu.edu/comparison/comp_hierarchy.jsp?root=509&depth=10&confidence=0.95) (1/1/9.05E-2)  »  »  »  »  »  »  »  genus [Maribacter](https://rdp.cme.msu.edu/comparison/comp_hierarchy.jsp?root=520&depth=10&confidence=0.95) (3/0/5.44E-4)  »  »  »  »  »  »  »  genus [Formosa](https://rdp.cme.msu.edu/comparison/comp_hierarchy.jsp?root=497&depth=10&confidence=0.95) (0/36/1.24E-2)  »  »  »  »  »  »  »  genus [Polaribacter](https://rdp.cme.msu.edu/comparison/comp_hierarchy.jsp?root=536&depth=10&confidence=0.95) (2/5/1.44E-1)  »  »  »  »  »  »  »  genus [Tenacibaculum](https://rdp.cme.msu.edu/comparison/comp_hierarchy.jsp?root=554&depth=10&confidence=0.95) (1/1/9.05E-2)  »  »  »  »  »  »  »  genus [Ulvibacter](https://rdp.cme.msu.edu/comparison/comp_hierarchy.jsp?root=555&depth=10&confidence=0.95) (2/0/4.24E-3)  »  »  »  »  »  »  »  genus [Flavobacterium](https://rdp.cme.msu.edu/comparison/comp_hierarchy.jsp?root=496&depth=10&confidence=0.95) (1/3/2.53E-1)  »  »  »  »  »  »  »  genus [Arenibacter](https://rdp.cme.msu.edu/comparison/comp_hierarchy.jsp?root=476&depth=10&confidence=0.95) (1/0/3.3E-2)  »  »  »  »  »  »  »  genus [Salegentibacter](https://rdp.cme.msu.edu/comparison/comp_hierarchy.jsp?root=543&depth=10&confidence=0.95) (1/0/3.3E-2)  »  »  »  »  »  »  »  genus [Persicivirga](https://rdp.cme.msu.edu/comparison/comp_hierarchy.jsp?root=533&depth=10&confidence=0.95) (1/0/3.3E-2)  »  »  »  »  »  »  »  [unclassified Flavobacteriaceae](https://rdp.cme.msu.edu/comparison/comp_hierarchy.jsp?root=-470&depth=10&confidence=0.95) (29/380/NA)  »  »  »  »  »  »  family [Cryomorphaceae](https://rdp.cme.msu.edu/comparison/comp_hierarchy.jsp?root=462&depth=10&confidence=0.95) (9/18/1.38E-3)  »  »  »  »  »  »  »  genus [Crocinitomix](https://rdp.cme.msu.edu/comparison/comp_hierarchy.jsp?root=464&depth=10&confidence=0.95) (6/0/1.15E-6)  »  »  »  »  »  »  »  genus [Owenweeksia](https://rdp.cme.msu.edu/comparison/comp_hierarchy.jsp?root=468&depth=10&confidence=0.95) (1/0/3.3E-2)  »  »  »  »  »  »  »  [unclassified Cryomorphaceae](https://rdp.cme.msu.edu/comparison/comp_hierarchy.jsp?root=-462&depth=10&confidence=0.95) (2/18/NA)  »  »  »  »  »  »  [unclassified "Flavobacteriales"](https://rdp.cme.msu.edu/comparison/comp_hierarchy.jsp?root=-459&depth=10&confidence=0.95) (1/122/NA)  »  »  »  »  [unclassified "Bacteroidetes"](https://rdp.cme.msu.edu/comparison/comp_hierarchy.jsp?root=-422&depth=10&confidence=0.95) (3/216/NA) |
| »  »  »  phylum ["Actinobacteria"](https://rdp.cme.msu.edu/comparison/comp_hierarchy.jsp?root=2&depth=10&confidence=0.95) (10/12/4.23E-6)  »  »  »  »  class [Actinobacteria](https://rdp.cme.msu.edu/comparison/comp_hierarchy.jsp?root=3&depth=10&confidence=0.95) (10/12/4.23E-6)  »  »  »  »  »  subclass [Actinobacteridae](https://rdp.cme.msu.edu/comparison/comp_hierarchy.jsp?root=16&depth=10&confidence=0.95) (4/7/2.53E-2)  »  »  »  »  »  »  order [Actinomycetales](https://rdp.cme.msu.edu/comparison/comp_hierarchy.jsp?root=17&depth=10&confidence=0.95) (4/7/2.53E-2)  »  »  »  »  »  »  »  suborder [Corynebacterineae](https://rdp.cme.msu.edu/comparison/comp_hierarchy.jsp?root=35&depth=10&confidence=0.95) (1/0/3.3E-2)  »  »  »  »  »  »  »  »  family [Dietziaceae](https://rdp.cme.msu.edu/comparison/comp_hierarchy.jsp?root=41&depth=10&confidence=0.95) (1/0/3.3E-2)  »  »  »  »  »  »  »  »  »  genus [Dietzia](https://rdp.cme.msu.edu/comparison/comp_hierarchy.jsp?root=42&depth=10&confidence=0.95) (1/0/3.3E-2)  »  »  »  »  »  »  »  suborder [Propionibacterineae](https://rdp.cme.msu.edu/comparison/comp_hierarchy.jsp?root=254&depth=10&confidence=0.95) (2/0/4.24E-3)  »  »  »  »  »  »  »  »  family [Nocardioidaceae](https://rdp.cme.msu.edu/comparison/comp_hierarchy.jsp?root=255&depth=10&confidence=0.95) (1/0/3.3E-2)  »  »  »  »  »  »  »  »  »  genus [Nocardioides](https://rdp.cme.msu.edu/comparison/comp_hierarchy.jsp?root=261&depth=10&confidence=0.95) (1/0/3.3E-2)  »  »  »  »  »  »  »  »  family [Propionibacteriaceae](https://rdp.cme.msu.edu/comparison/comp_hierarchy.jsp?root=264&depth=10&confidence=0.95) (1/0/3.3E-2)  »  »  »  »  »  »  »  »  »  genus [Tessaracoccus](https://rdp.cme.msu.edu/comparison/comp_hierarchy.jsp?root=279&depth=10&confidence=0.95) (1/0/3.3E-2)  »  »  »  »  »  »  »  suborder [Micrococcineae](https://rdp.cme.msu.edu/comparison/comp_hierarchy.jsp?root=95&depth=10&confidence=0.95) (1/1/9.05E-2)  »  »  »  »  »  »  »  »  family [Dermacoccaceae](https://rdp.cme.msu.edu/comparison/comp_hierarchy.jsp?root=119&depth=10&confidence=0.95) (0/1/1.52E0)  »  »  »  »  »  »  »  »  »  genus [Kytococcus](https://rdp.cme.msu.edu/comparison/comp_hierarchy.jsp?root=123&depth=10&confidence=0.95) (0/1/1.52E0)  »  »  »  »  »  »  »  »  family [Microbacteriaceae](https://rdp.cme.msu.edu/comparison/comp_hierarchy.jsp?root=154&depth=10&confidence=0.95) (1/0/3.3E-2)  »  »  »  »  »  »  »  »  »  genus [Agrococcus](https://rdp.cme.msu.edu/comparison/comp_hierarchy.jsp?root=156&depth=10&confidence=0.95) (1/0/3.3E-2)  »  »  »  »  »  »  »  [unclassified Actinomycetales](https://rdp.cme.msu.edu/comparison/comp_hierarchy.jsp?root=-17&depth=10&confidence=0.95) (0/6/NA)   »  »  »  »  »  subclass [Acidimicrobidae](https://rdp.cme.msu.edu/comparison/comp_hierarchy.jsp?root=4&depth=10&confidence=0.95) (6/5/5.04E-4)  »  »  »  »  »  »  order [Acidimicrobiales](https://rdp.cme.msu.edu/comparison/comp_hierarchy.jsp?root=5&depth=10&confidence=0.95) (6/5/5.04E-4)  »  »  »  »  »  »  »  suborder ["Acidimicrobineae"](https://rdp.cme.msu.edu/comparison/comp_hierarchy.jsp?root=6&depth=10&confidence=0.95) (6/5/5.04E-4)  »  »  »  »  »  »  »  »  family [Acidimicrobiaceae](https://rdp.cme.msu.edu/comparison/comp_hierarchy.jsp?root=7&depth=10&confidence=0.95) (1/4/3.48E-1)  »  »  »  »  »  »  »  »  »  genus [Ilumatobacter](https://rdp.cme.msu.edu/comparison/comp_hierarchy.jsp?root=11&depth=10&confidence=0.95) (1/4/3.48E-1)  »  »  »  »  »  »  »  »  [unclassified "Acidimicrobineae"](https://rdp.cme.msu.edu/comparison/comp_hierarchy.jsp?root=-6&depth=10&confidence=0.95) (5/1/NA) |
| »  »  »  phylum ["Proteobacteria"](https://rdp.cme.msu.edu/comparison/comp_hierarchy.jsp?root=841&depth=10&confidence=0.95) (168/779/1.98E-9)  »  »  »  »  class [Betaproteobacteria](https://rdp.cme.msu.edu/comparison/comp_hierarchy.jsp?root=1175&depth=10&confidence=0.95) (5/21/2.48E-1)  »  »  »  »  »  order [Nitrosomonadales](https://rdp.cme.msu.edu/comparison/comp_hierarchy.jsp?root=1318&depth=10&confidence=0.95) (1/0/3.3E-2)  »  »  »  »  »  »  family [Nitrosomonadaceae](https://rdp.cme.msu.edu/comparison/comp_hierarchy.jsp?root=1321&depth=10&confidence=0.95) (1/0/3.3E-2)  »  »  »  »  »  »  »  genus [Nitrosospira](https://rdp.cme.msu.edu/comparison/comp_hierarchy.jsp?root=1324&depth=10&confidence=0.95) (1/0/3.3E-2)  »  »  »  »  »  order [Burkholderiales](https://rdp.cme.msu.edu/comparison/comp_hierarchy.jsp?root=1176&depth=10&confidence=0.95) (2/7/2.54E-1)  »  »  »  »  »  »  family [Burkholderiales_incertae_sedis](https://rdp.cme.msu.edu/comparison/comp_hierarchy.jsp?root=1255&depth=10&confidence=0.95) (0/2/1.32E0)  »  »  »  »  »  »  »  genus [Aquabacterium](https://rdp.cme.msu.edu/comparison/comp_hierarchy.jsp?root=1256&depth=10&confidence=0.95) (0/2/1.32E0)  »  »  »  »  »  »  family [Comamonadaceae](https://rdp.cme.msu.edu/comparison/comp_hierarchy.jsp?root=1206&depth=10&confidence=0.95) (0/4/1.01E0)  »  »  »  »  »  »  »  genus [Acidovorax](https://rdp.cme.msu.edu/comparison/comp_hierarchy.jsp?root=1207&depth=10&confidence=0.95) (0/3/1.15E0)  »  »  »  »  »  »  »  [unclassified Comamonadaceae](https://rdp.cme.msu.edu/comparison/comp_hierarchy.jsp?root=-1206&depth=10&confidence=0.95) (0/1/NA)  »  »  »  »  »  »  family [Oxalobacteraceae](https://rdp.cme.msu.edu/comparison/comp_hierarchy.jsp?root=1240&depth=10&confidence=0.95) (1/1/9.05E-2)  »  »  »  »  »  »  »  genus [Herminiimonas](https://rdp.cme.msu.edu/comparison/comp_hierarchy.jsp?root=1244&depth=10&confidence=0.95) (0/1/1.52E0)  »  »  »  »  »  »  »  genus [Undibacterium](https://rdp.cme.msu.edu/comparison/comp_hierarchy.jsp?root=1251&depth=10&confidence=0.95) (1/0/3.3E-2)  »  »  »  »  »  »  family [Alcaligenaceae](https://rdp.cme.msu.edu/comparison/comp_hierarchy.jsp?root=1177&depth=10&confidence=0.95) (1/0/3.3E-2)  »  »  »  »  »  »  »  genus [Achromobacter](https://rdp.cme.msu.edu/comparison/comp_hierarchy.jsp?root=1178&depth=10&confidence=0.95) (1/0/3.3E-2)  »  »  »  »  »  order [Methylophilales](https://rdp.cme.msu.edu/comparison/comp_hierarchy.jsp?root=1279&depth=10&confidence=0.95) (2/1/1.53E-2)  »  »  »  »  »  »  family [Methylophilaceae](https://rdp.cme.msu.edu/comparison/comp_hierarchy.jsp?root=1280&depth=10&confidence=0.95) (2/1/1.53E-2)  »  »  »  »  »  »  »  [unclassified Methylophilaceae](https://rdp.cme.msu.edu/comparison/comp_hierarchy.jsp?root=-1280&depth=10&confidence=0.95) (2/1/NA)  »  »  »  »  »  [unclassified Betaproteobacteria](https://rdp.cme.msu.edu/comparison/comp_hierarchy.jsp?root=-1175&depth=10&confidence=0.95) (0/13/NA) |
| »  »  »  »  class [Deltaproteobacteria](https://rdp.cme.msu.edu/comparison/comp_hierarchy.jsp?root=1352&depth=10&confidence=0.95) (3/0/5.44E-4)  »  »  »  »  »  order [Bdellovibrionales](https://rdp.cme.msu.edu/comparison/comp_hierarchy.jsp?root=1353&depth=10&confidence=0.95) (3/0/5.44E-4)  »  »  »  »  »  »  family [Bacteriovoracaceae](https://rdp.cme.msu.edu/comparison/comp_hierarchy.jsp?root=1354&depth=10&confidence=0.95) (3/0/5.44E-4)  »  »  »  »  »  »  »  genus [Bacteriovorax](https://rdp.cme.msu.edu/comparison/comp_hierarchy.jsp?root=1355&depth=10&confidence=0.95) (2/0/4.24E-3)  »  »  »  »  »  »  »  [unclassified Bacteriovoracaceae](https://rdp.cme.msu.edu/comparison/comp_hierarchy.jsp?root=-1354&depth=10&confidence=0.95) (1/0/NA) |
| »  »  »  »  class [Gammaproteobacteria](https://rdp.cme.msu.edu/comparison/comp_hierarchy.jsp?root=1501&depth=10&confidence=0.95) (83/161/6E-14)  »  »  »  »  »  order [Aeromonadales](https://rdp.cme.msu.edu/comparison/comp_hierarchy.jsp?root=1507&depth=10&confidence=0.95) (1/0/3.3E-2)  »  »  »  »  »  »  family [Aeromonadaceae](https://rdp.cme.msu.edu/comparison/comp_hierarchy.jsp?root=1508&depth=10&confidence=0.95) (1/0/3.3E-2)  »  »  »  »  »  »  »  genus [Aeromonas](https://rdp.cme.msu.edu/comparison/comp_hierarchy.jsp?root=1509&depth=10&confidence=0.95) (1/0/3.3E-2)  »  »  »  »  »  order ["Enterobacteriales"](https://rdp.cme.msu.edu/comparison/comp_hierarchy.jsp?root=1616&depth=10&confidence=0.95) (1/0/3.3E-2)  »  »  »  »  »  »  family [Enterobacteriaceae](https://rdp.cme.msu.edu/comparison/comp_hierarchy.jsp?root=1617&depth=10&confidence=0.95) (1/0/3.3E-2)  »  »  »  »  »  »  »  genus [Citrobacter](https://rdp.cme.msu.edu/comparison/comp_hierarchy.jsp?root=1626&depth=10&confidence=0.95) (1/0/3.3E-2)  »  »  »  »  »  order [Xanthomonadales](https://rdp.cme.msu.edu/comparison/comp_hierarchy.jsp?root=1826&depth=10&confidence=0.95) (1/4/3.48E-1)  »  »  »  »  »  »  family [Xanthomonadaceae](https://rdp.cme.msu.edu/comparison/comp_hierarchy.jsp?root=1834&depth=10&confidence=0.95) (0/4/1.01E0)  »  »  »  »  »  »  »  genus [Stenotrophomonas](https://rdp.cme.msu.edu/comparison/comp_hierarchy.jsp?root=1851&depth=10&confidence=0.95) (0/4/1.01E0)  »  »  »  »  »  »  family [Sinobacteraceae](https://rdp.cme.msu.edu/comparison/comp_hierarchy.jsp?root=1827&depth=10&confidence=0.95) (1/0/3.3E-2)  »  »  »  »  »  »  »  [unclassified Sinobacteraceae](https://rdp.cme.msu.edu/comparison/comp_hierarchy.jsp?root=-1827&depth=10&confidence=0.95) (1/0/NA)  »  »  »  »  »  genus [Zhongshania](https://rdp.cme.msu.edu/comparison/comp_hierarchy.jsp?root=2826&depth=10&confidence=0.95) (1/0/3.3E-2)  »  »  »  »  »  order [Gammaproteobacteria_incertae_sedis](https://rdp.cme.msu.edu/comparison/comp_hierarchy.jsp?root=1856&depth=10&confidence=0.95) (6/0/1.15E-6)  »  »  »  »  »  »  genus [Thioprofundum](https://rdp.cme.msu.edu/comparison/comp_hierarchy.jsp?root=1877&depth=10&confidence=0.95) (1/0/3.3E-2)  »  »  »  »  »  »  genus [Marinicella](https://rdp.cme.msu.edu/comparison/comp_hierarchy.jsp?root=1864&depth=10&confidence=0.95) (1/0/3.3E-2)  »  »  »  »  »  »  genus [Sedimenticola](https://rdp.cme.msu.edu/comparison/comp_hierarchy.jsp?root=1869&depth=10&confidence=0.95) (1/0/3.3E-2)  »  »  »  »  »  »  genus [Porticoccus](https://rdp.cme.msu.edu/comparison/comp_hierarchy.jsp?root=1868&depth=10&confidence=0.95) (2/0/4.24E-3)  »  »  »  »  »  »  [unclassified Gammaproteobacteria_incertae_sedis](https://rdp.cme.msu.edu/comparison/comp_hierarchy.jsp?root=-1856&depth=10&confidence=0.95) (1/0/NA)  »  »  »  »  »  order [Pseudomonadales](https://rdp.cme.msu.edu/comparison/comp_hierarchy.jsp?root=1762&depth=10&confidence=0.95) (5/12/4.2E-2)  »  »  »  »  »  »  family [Moraxellaceae](https://rdp.cme.msu.edu/comparison/comp_hierarchy.jsp?root=1763&depth=10&confidence=0.95) (1/9/1.16E0)  »  »  »  »  »  »  »  genus [Acinetobacter](https://rdp.cme.msu.edu/comparison/comp_hierarchy.jsp?root=1764&depth=10&confidence=0.95) (0/4/1.01E0)  »  »  »  »  »  »  »  genus [Psychrobacter](https://rdp.cme.msu.edu/comparison/comp_hierarchy.jsp?root=1771&depth=10&confidence=0.95) (1/4/3.48E-1)  »  »  »  »  »  »  »  [unclassified Moraxellaceae](https://rdp.cme.msu.edu/comparison/comp_hierarchy.jsp?root=-1763&depth=10&confidence=0.95) (0/1/NA)  »  »  »  »  »  »  family [Pseudomonadaceae](https://rdp.cme.msu.edu/comparison/comp_hierarchy.jsp?root=1772&depth=10&confidence=0.95) (4/2/1.17E-3)  »  »  »  »  »  »  »  genus [Pseudomonas](https://rdp.cme.msu.edu/comparison/comp_hierarchy.jsp?root=1781&depth=10&confidence=0.95) (4/2/1.17E-3)  »  »  »  »  »  »  [unclassified Pseudomonadales](https://rdp.cme.msu.edu/comparison/comp_hierarchy.jsp?root=-1762&depth=10&confidence=0.95) (0/1/NA)  »  »  »  »  »  order [Alteromonadales](https://rdp.cme.msu.edu/comparison/comp_hierarchy.jsp?root=1520&depth=10&confidence=0.95) (11/4/4.42E-8)  »  »  »  »  »  »  family [Shewanellaceae](https://rdp.cme.msu.edu/comparison/comp_hierarchy.jsp?root=1557&depth=10&confidence=0.95) (1/0/3.3E-2)  »  »  »  »  »  »  »  genus [Shewanella](https://rdp.cme.msu.edu/comparison/comp_hierarchy.jsp?root=1558&depth=10&confidence=0.95) (1/0/3.3E-2)  »  »  »  »  »  »  family [Pseudoalteromonadaceae](https://rdp.cme.msu.edu/comparison/comp_hierarchy.jsp?root=1551&depth=10&confidence=0.95) (2/0/4.24E-3)  »  »  »  »  »  »  »  genus [Pseudoalteromonas](https://rdp.cme.msu.edu/comparison/comp_hierarchy.jsp?root=1553&depth=10&confidence=0.95) (2/0/4.24E-3)  »  »  »  »  »  »  family [Alteromonadaceae](https://rdp.cme.msu.edu/comparison/comp_hierarchy.jsp?root=1521&depth=10&confidence=0.95) (4/3/2.79E-3)  »  »  »  »  »  »  »  genus [Glaciecola](https://rdp.cme.msu.edu/comparison/comp_hierarchy.jsp?root=1528&depth=10&confidence=0.95) (1/0/3.3E-2)  »  »  »  »  »  »  »  genus [Haliea](https://rdp.cme.msu.edu/comparison/comp_hierarchy.jsp?root=1529&depth=10&confidence=0.95) (1/3/2.53E-1)  »  »  »  »  »  »  »  genus [Marinobacter](https://rdp.cme.msu.edu/comparison/comp_hierarchy.jsp?root=1531&depth=10&confidence=0.95) (2/0/4.24E-3)  »  »  »  »  »  »  family [Colwelliaceae](https://rdp.cme.msu.edu/comparison/comp_hierarchy.jsp?root=1539&depth=10&confidence=0.95) (2/1/1.53E-2)  »  »  »  »  »  »  »  genus [Colwellia](https://rdp.cme.msu.edu/comparison/comp_hierarchy.jsp?root=1540&depth=10&confidence=0.95) (2/0/4.24E-3)  »  »  »  »  »  »  »  [unclassified Colwelliaceae](https://rdp.cme.msu.edu/comparison/comp_hierarchy.jsp?root=-1539&depth=10&confidence=0.95) (0/1/NA)  »  »  »  »  »  »  family [Idiomarinaceae](https://rdp.cme.msu.edu/comparison/comp_hierarchy.jsp?root=1545&depth=10&confidence=0.95) (2/0/4.24E-3)  »  »  »  »  »  »  »  genus [Idiomarina](https://rdp.cme.msu.edu/comparison/comp_hierarchy.jsp?root=1546&depth=10&confidence=0.95) (2/0/4.24E-3)  »  »  »  »  »  order [Oceanospirillales](https://rdp.cme.msu.edu/comparison/comp_hierarchy.jsp?root=1694&depth=10&confidence=0.95) (15/2/1.29E-12)  »  »  »  »  »  »  family [Halomonadaceae](https://rdp.cme.msu.edu/comparison/comp_hierarchy.jsp?root=1705&depth=10&confidence=0.95) (3/0/5.44E-4)  »  »  »  »  »  »  »  genus [Halomonas](https://rdp.cme.msu.edu/comparison/comp_hierarchy.jsp?root=1711&depth=10&confidence=0.95) (3/0/5.44E-4)  »  »  »  »  »  »  family [Alcanivoracaceae](https://rdp.cme.msu.edu/comparison/comp_hierarchy.jsp?root=1695&depth=10&confidence=0.95) (6/0/1.15E-6)  »  »  »  »  »  »  »  genus [Kangiella](https://rdp.cme.msu.edu/comparison/comp_hierarchy.jsp?root=1698&depth=10&confidence=0.95) (1/0/3.3E-2)  »  »  »  »  »  »  »  genus [Alcanivorax](https://rdp.cme.msu.edu/comparison/comp_hierarchy.jsp?root=1696&depth=10&confidence=0.95) (5/0/8.97E-6)  »  »  »  »  »  »  family [Oceanospirillaceae](https://rdp.cme.msu.edu/comparison/comp_hierarchy.jsp?root=1721&depth=10&confidence=0.95) (6/0/1.15E-6)  »  »  »  »  »  »  »  genus [Thalassolituus](https://rdp.cme.msu.edu/comparison/comp_hierarchy.jsp?root=1737&depth=10&confidence=0.95) (2/0/4.24E-3)  »  »  »  »  »  »  »  genus [Neptuniibacter](https://rdp.cme.msu.edu/comparison/comp_hierarchy.jsp?root=1727&depth=10&confidence=0.95) (2/0/4.24E-3)  »  »  »  »  »  »  »  genus [Marinomonas](https://rdp.cme.msu.edu/comparison/comp_hierarchy.jsp?root=1725&depth=10&confidence=0.95) (1/0/3.3E-2)  »  »  »  »  »  »  »  genus [Oleispira](https://rdp.cme.msu.edu/comparison/comp_hierarchy.jsp?root=1734&depth=10&confidence=0.95) (1/0/3.3E-2)  »  »  »  »  »  »  [unclassified Oceanospirillales](https://rdp.cme.msu.edu/comparison/comp_hierarchy.jsp?root=-1694&depth=10&confidence=0.95) (0/2/NA)  »  »  »  »  »  order [Thiotrichales](https://rdp.cme.msu.edu/comparison/comp_hierarchy.jsp?root=1790&depth=10&confidence=0.95) (4/0/6.99E-5)  »  »  »  »  »  »  family [Piscirickettsiaceae](https://rdp.cme.msu.edu/comparison/comp_hierarchy.jsp?root=1793&depth=10&confidence=0.95) (4/0/6.99E-5)  »  »  »  »  »  »  »  genus [Cycloclasticus](https://rdp.cme.msu.edu/comparison/comp_hierarchy.jsp?root=1794&depth=10&confidence=0.95) (1/0/3.3E-2)  »  »  »  »  »  »  »  genus [Methylophaga](https://rdp.cme.msu.edu/comparison/comp_hierarchy.jsp?root=1796&depth=10&confidence=0.95) (3/0/5.44E-4)  »  »  »  »  »  [unclassified Gammaproteobacteria](https://rdp.cme.msu.edu/comparison/comp_hierarchy.jsp?root=-1501&depth=10&confidence=0.95) (38/139/NA) |
| »  »  »  »  class [Alphaproteobacteria](https://rdp.cme.msu.edu/comparison/comp_hierarchy.jsp?root=842&depth=10&confidence=0.95) (75/529/7.11E-1)  »  »  »  »  »  order [Rhodobacterales](https://rdp.cme.msu.edu/comparison/comp_hierarchy.jsp?root=978&depth=10&confidence=0.95) (26/318/1.38E-3)  »  »  »  »  »  »  family [Rhodobacteraceae](https://rdp.cme.msu.edu/comparison/comp_hierarchy.jsp?root=979&depth=10&confidence=0.95) (26/318/1.38E-3)  »  »  »  »  »  »  »  genus [Phaeobacter](https://rdp.cme.msu.edu/comparison/comp_hierarchy.jsp?root=1023&depth=10&confidence=0.95) (1/0/3.3E-2)  »  »  »  »  »  »  »  genus [Octadecabacter](https://rdp.cme.msu.edu/comparison/comp_hierarchy.jsp?root=1016&depth=10&confidence=0.95) (1/0/3.3E-2)  »  »  »  »  »  »  »  genus [Sulfitobacter](https://rdp.cme.msu.edu/comparison/comp_hierarchy.jsp?root=1056&depth=10&confidence=0.95) (5/0/8.97E-6)  »  »  »  »  »  »  »  genus [Litoreibacter](https://rdp.cme.msu.edu/comparison/comp_hierarchy.jsp?root=1001&depth=10&confidence=0.95) (1/1/9.05E-2)  »  »  »  »  »  »  »  genus [Loktanella](https://rdp.cme.msu.edu/comparison/comp_hierarchy.jsp?root=1003&depth=10&confidence=0.95) (2/0/4.24E-3)  »  »  »  »  »  »  »  genus [Roseovarius](https://rdp.cme.msu.edu/comparison/comp_hierarchy.jsp?root=1042&depth=10&confidence=0.95) (1/2/1.66E-1)  »  »  »  »  »  »  »  genus [Ahrensia](https://rdp.cme.msu.edu/comparison/comp_hierarchy.jsp?root=981&depth=10&confidence=0.95) (1/0/3.3E-2)  »  »  »  »  »  »  »  [unclassified Rhodobacteraceae](https://rdp.cme.msu.edu/comparison/comp_hierarchy.jsp?root=-979&depth=10&confidence=0.95) (14/315/NA)  »  »  »  »  »  order [Rhizobiales](https://rdp.cme.msu.edu/comparison/comp_hierarchy.jsp?root=870&depth=10&confidence=0.95) (8/0/1.9E-8)  »  »  »  »  »  »  family [Brucellaceae](https://rdp.cme.msu.edu/comparison/comp_hierarchy.jsp?root=898&depth=10&confidence=0.95) (1/0/3.3E-2)  »  »  »  »  »  »  »  genus [Ochrobactrum](https://rdp.cme.msu.edu/comparison/comp_hierarchy.jsp?root=903&depth=10&confidence=0.95) (1/0/3.3E-2)  »  »  »  »  »  »  family [Phyllobacteriaceae](https://rdp.cme.msu.edu/comparison/comp_hierarchy.jsp?root=940&depth=10&confidence=0.95) (3/0/5.44E-4)  »  »  »  »  »  »  »  genus [Hoeflea](https://rdp.cme.msu.edu/comparison/comp_hierarchy.jsp?root=945&depth=10&confidence=0.95) (3/0/5.44E-4)  »  »  »  »  »  »  family [Hyphomicrobiaceae](https://rdp.cme.msu.edu/comparison/comp_hierarchy.jsp?root=908&depth=10&confidence=0.95) (3/0/5.44E-4)  »  »  »  »  »  »  »  genus [Devosia](https://rdp.cme.msu.edu/comparison/comp_hierarchy.jsp?root=914&depth=10&confidence=0.95) (1/0/3.3E-2)  »  »  »  »  »  »  »  genus [Pelagibacterium](https://rdp.cme.msu.edu/comparison/comp_hierarchy.jsp?root=2918&depth=10&confidence=0.95) (1/0/3.3E-2)  »  »  »  »  »  »  »  [unclassified Hyphomicrobiaceae](https://rdp.cme.msu.edu/comparison/comp_hierarchy.jsp?root=-908&depth=10&confidence=0.95) (1/0/NA)  »  »  »  »  »  »  family ["Aurantimonadaceae"](https://rdp.cme.msu.edu/comparison/comp_hierarchy.jsp?root=871&depth=10&confidence=0.95) (1/0/3.3E-2)  »  »  »  »  »  »  »  genus [Aurantimonas](https://rdp.cme.msu.edu/comparison/comp_hierarchy.jsp?root=872&depth=10&confidence=0.95) (1/0/3.3E-2)  »  »  »  »  »  order [Sphingomonadales](https://rdp.cme.msu.edu/comparison/comp_hierarchy.jsp?root=1151&depth=10&confidence=0.95) (3/2/6.57E-3)  »  »  »  »  »  »  family [Sphingomonadaceae](https://rdp.cme.msu.edu/comparison/comp_hierarchy.jsp?root=1158&depth=10&confidence=0.95) (1/1/9.05E-2)  »  »  »  »  »  »  »  [unclassified Sphingomonadaceae](https://rdp.cme.msu.edu/comparison/comp_hierarchy.jsp?root=-1158&depth=10&confidence=0.95) (1/1/NA)  »  »  »  »  »  »  family [Erythrobacteraceae](https://rdp.cme.msu.edu/comparison/comp_hierarchy.jsp?root=1152&depth=10&confidence=0.95) (2/1/1.53E-2)  »  »  »  »  »  »  »  genus [Porphyrobacter](https://rdp.cme.msu.edu/comparison/comp_hierarchy.jsp?root=1157&depth=10&confidence=0.95) (0/1/1.52E0)  »  »  »  »  »  »  »  genus [Erythrobacter](https://rdp.cme.msu.edu/comparison/comp_hierarchy.jsp?root=1155&depth=10&confidence=0.95) (1/0/3.3E-2)  »  »  »  »  »  »  »  [unclassified Erythrobacteraceae](https://rdp.cme.msu.edu/comparison/comp_hierarchy.jsp?root=-1152&depth=10&confidence=0.95) (1/0/NA)  »  »  »  »  »  order [Caulobacterales](https://rdp.cme.msu.edu/comparison/comp_hierarchy.jsp?root=843&depth=10&confidence=0.95) (2/5/1.44E-1)  »  »  »  »  »  »  family [Caulobacteraceae](https://rdp.cme.msu.edu/comparison/comp_hierarchy.jsp?root=844&depth=10&confidence=0.95) (0/5/8.77E-1)  »  »  »  »  »  »  »  genus [Brevundimonas](https://rdp.cme.msu.edu/comparison/comp_hierarchy.jsp?root=846&depth=10&confidence=0.95) (0/5/8.77E-1)  »  »  »  »  »  »  family [Hyphomonadaceae](https://rdp.cme.msu.edu/comparison/comp_hierarchy.jsp?root=849&depth=10&confidence=0.95) (2/0/4.24E-3)  »  »  »  »  »  »  »  genus [Hyphomonas](https://rdp.cme.msu.edu/comparison/comp_hierarchy.jsp?root=853&depth=10&confidence=0.95) (1/0/3.3E-2)  »  »  »  »  »  »  »  genus [Maricaulis](https://rdp.cme.msu.edu/comparison/comp_hierarchy.jsp?root=856&depth=10&confidence=0.95) (1/0/3.3E-2)  »  »  »  »  »  order [Rhodospirillales](https://rdp.cme.msu.edu/comparison/comp_hierarchy.jsp?root=1069&depth=10&confidence=0.95) (5/2/1.99E-4)  »  »  »  »  »  »  family [Rhodospirillaceae](https://rdp.cme.msu.edu/comparison/comp_hierarchy.jsp?root=1101&depth=10&confidence=0.95) (5/2/1.99E-4)  »  »  »  »  »  »  »  genus [Thalassospira](https://rdp.cme.msu.edu/comparison/comp_hierarchy.jsp?root=1127&depth=10&confidence=0.95) (1/0/3.3E-2)  »  »  »  »  »  »  »  genus [Nisaea](https://rdp.cme.msu.edu/comparison/comp_hierarchy.jsp?root=1113&depth=10&confidence=0.95) (1/0/3.3E-2)  »  »  »  »  »  »  »  [unclassified Rhodospirillaceae](https://rdp.cme.msu.edu/comparison/comp_hierarchy.jsp?root=-1101&depth=10&confidence=0.95) (3/2/NA)  »  »  »  »  »  family [SAR11](https://rdp.cme.msu.edu/comparison/comp_hierarchy.jsp?root=2940&depth=10&confidence=0.95) (20/166/3.73E-1)  »  »  »  »  »  »  genus [Candidatus Pelagibacter](https://rdp.cme.msu.edu/comparison/comp_hierarchy.jsp?root=2941&depth=10&confidence=0.95) (20/166/3.73E-1)  »  »  »  »  »  [unclassified Alphaproteobacteria](https://rdp.cme.msu.edu/comparison/comp_hierarchy.jsp?root=-842&depth=10&confidence=0.95) (11/36/NA)  »  »  »  »  [unclassified "Proteobacteria"](https://rdp.cme.msu.edu/comparison/comp_hierarchy.jsp?root=-841&depth=10&confidence=0.95) (2/68/NA) |
